# FXR-mediated inhibition of autophagy contributes to FA-induced TG accumulation and accordingly reduces FA-induced lipotoxicity

**DOI:** 10.1186/s12964-020-0525-1

**Published:** 2020-03-20

**Authors:** Kun Wu, Tao Zhao, Christer Hogstrand, Yi-Chuang Xu, Shi-Cheng Ling, Guang-Hui Chen, Zhi Luo

**Affiliations:** 1 Key Laboratory of Freshwater Animal Breeding, Ministry of Agriculture of P.R.C., Fishery College, Huazhong Agricultural University, Wuhan, 430070 China; 2 Diabetes and Nutritional Sciences Division, School of Medicine, King’s College London, London, UK; 3 Laboratory for Marine Fisheries Science and Food Production Processes, Qingdao National Laboratory for Marine Science and Technology, Qingdao, 266237 China

**Keywords:** Lipid metabolism, RNA-seq transcriptome, Autophagy, FXR, Lipotoxicity

## Abstract

**Background:**

Excessive dietary fat intake induces lipid deposition and contributes to the progress of nonalcoholic fatty liver disease (NAFLD). However, the underlying mechanisms are still unclear.

**Methods:**

Yellow catfish were given two experimental diets with dietary lipid levels of 11.3 and 15.4%, respectively, for 56 days, and the contents of triglyceride (TG), nonesterified free fatty acids (NEFA) and bile acid (BA), RNA-seq, enzymatic activities and mRNA expression were deteremined in the liver tissues. Hepatocytes from yellow catfish liver tissues were isolated and cultured. Fatty acids (FA) (palmitic acid: OA, oleic acid =1:1), pathway inhibitors (MA, autophagy inhibitor; guggulsterone, FXR inhibitor) and agonist (rapamyicn, autophagy agonist; GW4064, FXR agonist) were used to incubate the cells. TG and NEFA contents, ultrastructural observation, autophagic vesicles and intracellular LD,apoptosis,western blot and Co-IP, and Immunofluorescence analysis, enzymatic activities and Q-PCR were decided.

**Results:**

Using RNA sequencing, we found that high fat diets induced changes in expression of many genes associated with the pathways of lipid metabolism and autophagy. The mRNA profiles of the differentially expressed genes (DEG) indicated that high dietary fat-induced lipid deposition was predominantly influenced by the inhibition of autophagy. Using primary hepatocytes, we found that fatty acids (FA) suppressed autophagy, which in turn reduced cellular free FA level by decreasing triglyceride (TG) breakdown. Moreover, our study indicated that farnesoid X receptor (FXR)-cyclic AMP-responsive element-binding protein (CREB) axis was the pivotal physiological switch regulating FA-induced changes of autophagy and lipid metabolism, which represented cellular defenses against FA-induced lipotoxicity.

**Conclusion:**

This discovery may provide new targets for treating pathological changes involved in the dysfunction of autophagy and metabolism, including NAFLD.

Video Abstract

**Graphical abstract:**

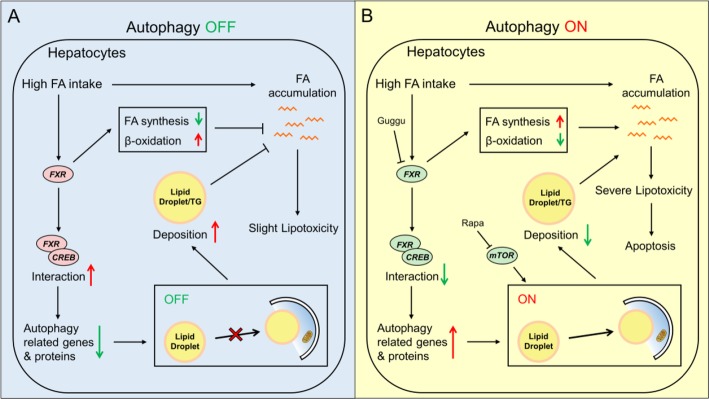

## Background

Obesity is a main risk factor for lots of chronic diseases, including type 2 diabetes, hypertension and cancer [1]. High dietary fat intake results in the obesity in mammals [2, 3]. The liver is the main organ for nutrient metabolism and plays a crucial role in obesity-related disorders and diabetes. Chronic and high lipid intake resulted in the occurrence of nonalcoholic fatty liver disease (NAFLD) that was related to the accumulation of triglyceride (TG) in liver tissues [4]. However, the underlying molecular mechanisms are still unknown.

Autophagy is an intracellular degradative system for cytosolic components, long-lived proteins and damaged organelles, and helps to maintain intracellular homeostasis under starvation, oxidative stress and/ or nutrient-rich environment [5, 6]. Autophagy also plays a critical function in regulating lipid metabolism because it degrades lipid droplets (LDs) and facilitates lipolysis [7]. The inhibition of lipophagy causes the up-regulation of TG contents in hepatocytes [8]. Disruption of autophagy is associated with lots of metabolic disorders, including obesity, liver injuries and NAFLD [8, 9]. These findings indicate that defective lipophagy or damaged autophagy underlies the progress of several metabolic disease, including NAFLD. However, how autophagy and/or lipophagy influencing these progresses is not well understood.

Many signaling molecules and pathways modulate autophagic activity. Mammalian target of rapamycin (mTOR) cuts off autophagy when animals possess enough growth factors and nutrients, and accordingly represents a suppressed signal for autophagy. Recently, studies suggested that the activation of bile acid (BA) receptor farnesoid X receptor (FXR) strongly suppressed autophagy in the liver during the fed-stage of mice, and this kind of the inhibition was independent of the mTOR pathway [10, 11]. Thus, since FXR is an important sensing regulator under the fed status, we hypothesize that FXR represses the autophagy under the high fat feeding.

Fish are the largest population among vertebrates. During the evolution, fish were considered to experience the fish-specific genome duplication event (FSGD). By analyzing whole-genome sequence information, Gong et al. [12] found the FSGD in yellow catfish *Pelteobagrus fulvidraco*, an important freshwater omnivorous fish in China and other countries. Some duplicated genes evolve new functions that in turn result in novel regulatory mechanism. Therefore, using yellow catfish as a model, we hope to find some novel regulatory mechanism of metabolism. Moreover, our previous study found that yellow catfish frequently exhibited excessive hepatic lipid deposition and severe fatty liver syndrome, which have an adverse effect on its health. Given the potential role of lipid in autophagy and the role of autophagy in lipid metabolism, we hypothesize that autophagy mediates the lipid-induced changes in lipid metabolism. Our study suggests that the activation of FXR-CREB (cyclic AMP-responsive element-binding protein) pathway inhibits autophagy and regulates lipid metabolism, which helps maintain cellular FA balance and protects the cells against lipotoxicity.

## Materials and methods

The present study consisted of two experiments. The experiments complied with the ethical guidelines of Huazhong Agricultural University (HZAU) for the use of experimental animals and cells, and were approved by the Ethical Committee of HZAU.

### In vivo studies: experimental protocols, sample collections and analysis

#### Experimental diets and fish culture

Two experimental diets were formulated with dietary lipid levels of 11.3 and 15.4%, respectively (Additional file 1: Supplemental Table S1). Soybean oil and fish oil were added in the diet at a ratio of 1: 1. 11.3% of dietary lipid represents the normal level in the commercial diet for yellow catfish and 15.4% of dietary lipid was higher than those in the commercial diet for the fish species. Each diet was fed to three tanks of yellow catfish (body weight: 3.8 ± 0.2 g/fish). Yellow catfish were fed to satiation twice daily. The experiment continued for 56 days.

At the end of the feeding experiment, yellow catfish were fasted for 24 h to avoid prandial effects. Yellow catfish were euthanized with tricaine methanesulfonate (100 mg/l), weighed in bulk and dissected to obtain liver tissues on ice. Samples were removed, frozen in liquid nitrogen and then stored at − 80 °C freezer for the subsequent determination of the contents of TG, nonesterified free fatty acids (NEFA) and bile acid (BA), enzymatic activities and mRNA expression.

#### Measurements of TG, NEFA and BA

The liver tissues were homogenized in the ice-cold buffers (0.25 M sucrose, 0.02 M Tris-HCl, 0.1 M sodium fluoride, 2 mM EDTA, 0.01 M β-mercapto-ethanol, 0.5 mM phenylmethyl sulfonyl fluoride, pH 7.4). The homogenates were then centrifuged at 20,000 g for 30 min at 4 °C, and the supernatant was used to measure the contents of TG, NEFA and BA by the commercial kits. The kits were purchased from Nanjing Jian Cheng Bioengineering Institute (Nanjing, China).

#### Transcriptome sequencing, assembly and annotation

We randomly selected three fish from each tank for RNA isolation. Trizol reagents (Invitrogen, USA) were used for RNA isolation. In total, six libraries from two groups (three tanks per treatment, three equivalent quantities of RNA from the same experimental tank were incorporated into one pool) were constructed and sequenced in two lanes with read length 100 bp on the BGISEQ-500 platform (Beijing Genome Institute, BGI, Shenzhen, China) [13].

After filtering adaptor sequences and low-quality sequences, we obtained the clean reads from these raw data. Then, we used the assembling program Trinity to assemble the clear reads into unigenes [14]. The BLASTx alignment (e-value < 10^− 5^) was used to annotate transcripts over 200 bp, based on databases of non-redundant nucleotide (Nt), NR (non-redundant protein sequence), COG (Clusters of Orthologous Groups), KEGG (Kyoto Encyclopedia of Genes and Genomes) and Swiss-Prot. Blast2GO [15] and WEGO [16] were used to perform GO (Gene Ontology) annotation and gene classification, respectively. Thus, we approximately generated 502 million reads, and every sample had 66.17 to 66.77 million clean reads (Additional file 2: Supplemental Table S2). After assembly, we obtained 69,307 unigenes. Their total length was 89,105,992 bp, the mean length 1285 bp, and the N50 length was 2492 bp (Additional file 3: Supplemental Table S3). We then searched all unigene sequences in public databases of NR, NT, GO, COG, KEGG, Swissprot and Interpro (Additional file 4: Supplemental Table S4). All of these reads have been submitted to the Sequence Read Archive at NCBI database (Accession Number: PRJNA49626).

#### Analysis of differentially expressed genes (DEG) and quantitative real-time PCR (Q-PCR) validation

The fragments per kilobase of transcript per million mapped reads (FPKM) method were used to calculate the mRNA levels of genes [17]. Noiseq method (probability ≥0.8 & log_2_fold-change > 1) was used to screen DEGs [18]. All these DEGs were mapped to the database of KEGG and GO for pathway and GO enrichment analysis.

We randomly selected twelve candidate genes for Q-PCR validation. The Q-PCR was performed on the qTOWER 2.0 (Analytik Jena, Germany) using Q-PCR kit (TaKaRa, Japan). The thermal cycling parameters were followed: 40 cycles of 5 s at 95 °C, 30s at 57 °C and 30s at 72 °C. We selected eight housekeeping genes (HPRT, β-actin, B2M, GAPDH, TUBA, ELFA, RPL7 and TBP) to determine their transcription stability. GeNorm [19] analysis indicated that β-actin and TBP were the most stable housekeeping genes at transcript level under the present in vivo conditions. Accordingly, we normalized the mRNA levels to the geometric mean of β-actin and TBP. The 2^-ΔΔCt^ method was used to calculate the fold changes of relative expression levels [20]. Primers for Q-PCR are given in Additional file 5: Supplemental Table S5.

### In vitro studies

#### Hepatocytes culture and treatments

We isolate hepatocytes from yellow catfish liver tissues according to our recent publication [21]. The isolated cells were seeded onto 25 cm^2^ plates (1 × 10^6^ cells/mL) and placed in an incubator at 28 °C and 0.5% CO_2_ (Sanyo, Japan). Two pathway inhibitors and two agonist were used in the in vitro trials. They are rapamycin (RM, autophagy agonist, Selleck, USA), 3-methyladenine (MA, autophagy inhibitor, Selleck, USA), GW4064 (GW, FXR agonist, Selleck, USA) and guggulsterone (GS, FXR inhibitor, Sigma, USA), respectively. Treatment groups were designed as follows: control, 0.5 mM FA (PA, palmitic acid: OA, oleic acid =1:1), 20 nM rapamyicn (RM), 5 mM MA, 20 μM guggulsterone (GS), 1 μM GW4064 (GW), 0.5 mM FA + 20 nM rapamycin (FA + RM), 0.5 mM FA + 5 mM MA, 0.5 mM FA + 1 μM GW4064 (FA + GW) and 0.5 mM FA + 20 μM guggulsterone (FA + GS), respectively. Each treatment had three replicates. Based on our pilot trials, the FA level was set at a concentration that did not adversely affect cell viability. We selected the inhibitors/agonist concentrations based on our other pilot trials and to previous reports [22, 23]. The cells were collected at 48 h for the analyses described below.

##### Cell viability, TG and NEFA contents

We used the tetrazolium dye (MTT) to assay cell viability. The contents of TG and NEFA were determined, based on the protocols mentioned above.

#### Ultrastructural observation

For the ultrastructural analysis, glutaraldehyde-fixed specimens were post-fixed in aqueous osmium tetroxide. The specimens were then dehydrated with graded ethanol, embedded in Epon and prepared for transmission electron microscope (TEM) (Tecnai G220TWIN, FEI company, USA) observation.

#### Detection of autophagic vesicles and intracellular LD

Autophagic vesicles and intracellular LD were detected based on the protocols of Klionsky et al. [5] as described in our previous study [24]. For detection of autophagic vesicles, cells were incubated with 1 mM acridine orange (AO, Sigma, USA), 50 mM monodansylcadaverine (MDC, Sigma, USA), or 50 nM LysoTracker Red (Sigma, USA) for 30 min. Then, they were washed three times in the ice-cold PBS. For intracellular LD staining, cells were incubated with 5 mg/ml Bodipy (Invitrogen, USA) for 30 min, and then washed three times in the ice-cold PBS. Fluorescence was imaged using laser scanning confocal microscopy (Leica, German) and flow cytometry (Beckman, USA) was used to determine fluorescence intensities.

#### Determination of apoptosis

The oxidative stress and apoptosis analysis were performed according to the methods described in our recent publication [13]. We used Annexin V-FITC and propidium iodide (PI) to measure the apoptotic cells. The commercial kit (Nanjing Jian Cheng Bioengineering Institute, Nanjing, China) was used to measure the caspase 3 activity. The flow cytometry (Beckman, USA) was used to measure the fluorescence intensity. The wavelengths of excitation and emission were 490 nm and 525 nm, respectively.

#### Western blot and co-IP

Western blot analysis followed the protocols in our recent study [24]. Briefly, hepatocytes were lysed in RIPA buffer (Sigma, USA). Equal amounts of protein were separated on 10% SDS-PAGE, transferred onto PVDF membranes, and then blocked with 8% (w/v) dry milk. After that, the membranes were incubated with primary antibodies as follows: rabbit anti-LC3B (Abcam, ab48394, USA), rabbit anti-Beclin1 (Abcam, ab62557, USA), rabbit anti-SQSTM1/p62 (Abcam, ab91526, USA), rabbit anti-FXR (Abcam, ab155124, USA), rabbit anti-CREB1 (Abcam, ab31387, USA) and anti-GAPDH (Abcam, ab9485, USA) overnight at 4 °C. Then, HRP-conjugated anti-rabbit secondary antibody (CST, USA) was used to probe with. Finally, the protein bands were visualized with enhanced chemiluminescent (ECL) and quantified by Image J software.

Coimmunoprecipitation (Co-IP) analysis followed the protocols described in Hsieh et al. [25]. Briefly, hepatocytes were washed in ice-cold PBS and lysed in Co-IP buffer containing protease inhibitors. Lysates were centrifuged at 4000 g for 10 min at 4 °C. Rabbit anti-FXR or anti-CREB1 was used to incubate the clarified lysates for overnight at 4 °C. The operations were followed by the addition of protein A-agarose beads prewashed in Co-IP buffer for an additional 1 h. Then, we washed beads three times in Co-IP buffer and one time in PBS. SDS-sample buffer was used to elute proteins bound to the beads. Then were analysed by SDS-PAGE and immunoblot.

#### Immunofluorescence analysis

Hepatocytes were grown on cover slides in a 12-well plate. After the treatment, hepatocytes were fixed in 4% paraformaldehyde. They were then blocked in 5% bovine serum albumin (BSA) diluted with 0.3% Triton X-100. The hepatocytes were incubated with anti-LC3B primary antibody (Abcam, USA) overnight at 4 °C. After washing thrice, the cells were incubated with a Goat Anti-Rabbit IgG (Sigma, USA) secondary antibody in the darkness under room temperature for 60 min. After staining with DAPI in the dark for 5 min, the hepatocytes were examined using a laser scanning confocal microscope (Leica, German).

#### Enzymatic activities and Q-PCR

Activities of lipid metabolism-related enzymes, such as malic enzyme (ME), isocitrate dehydrogenase (ICDH), glucose-6-phosphate dehydrogenase (G6PD), 6-phosphogluconate dehydrogenase (6PGD), fatty acid synthase (FAS), and carnitine palmitoyl transferases I (CPT I), were analyzed, as described in our recent publication [21]. One unit of enzyme activity, expressed as mU mg^− 1^ soluble protein, was defined as 1 μM of substrate converted to product per minute at 28 °C.

We performed Q-PCR in the in vitro experiment, based on the methods mentioned above. Under the experimental conditions in vitro, we selected the two most stable genes (TBP and TUBA) from eight housekeeping genes (β-actin, HPRT, B2M, GAPDH, RPL7, ELFA, TBP and TUBA) according to geNorm software. Primers were given in Additional file 5: Supplemental Table S5.

### Statistical analysis

We used SPSS 19.0 software for statistical analysis. Data were presented as means ± standard error of means (SEM). Before the statistical analysis, the Kolmogorov-Smirnov test was used to evaluate the data for normality of distribution. The homogeneity of variances was tested by the Bartlett’s test. Data were analyzed with one-way ANOVA and Student’s t-test where appropriate. The differences were considered to be statistically significant at *P* < 0.05.

## Results

### In vivo study

#### RNA-seq analysis and validation of DEGs

Our study identified 3882 unigenes as DEGs between the control and high-fat group, including 2283 up- and 1599 down-regulated genes (Additional file 7: Supplemental Fig. S1). GO (Additional file 8: Supplemental Fig. S2) and KEGG (Additional file 9: Supplemental Fig. S3) database were used to identify the function of DEGs. We found that high-fat diet had extensive influences on various biological pathways, including autophagy, lipolytic and glycolytic pathways.

For the validation of DEGs, we selected 12 DEGs (8 up- and 4 down-regulated DEGs) for Q-PCR analysis. Among the 12 genes, 11 transcript exhibited similar trends between RNA-seq and Q-PCR (Additional file 10: Supplemental Fig. S4). The correlation coefficient between Q-PCR and RNA-seq results was 0.903 (*p* < 0.001), confirming the validity of the results.

#### High-fat diet induced hepatic lipid accumulation

**Fig. 1:**
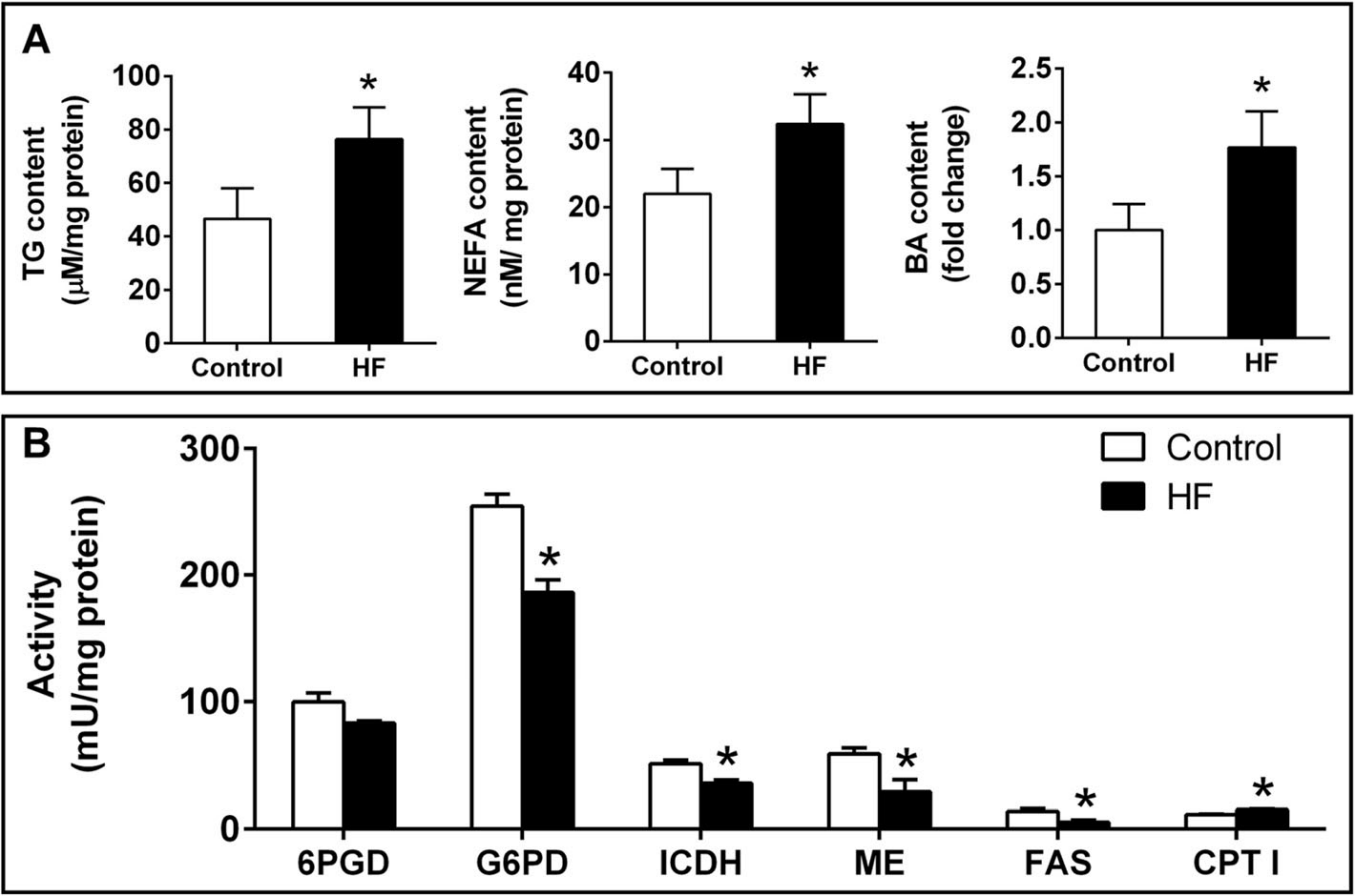
Effect of dietary fat levels on lipid metabolism, oxidative stress and caspase 3 activity in the liver of *P. fulvidraco*. **a** The contents of TG, NEFA and BA. **b** Activities of enzymes involved in lipid metabolism. HF: high dietary fat. Values are means ± SEM (*n* = 3). Asterisks (*) indicate significant differences between control and high-fat group

As expected, compared to the control, high-fat diet increased the contents of TG, NFFA and BA (Fig. 1a), but did not significantly affect caspase 3 activity (Fig. 1b). In addition, high-fat diet significantly increased CPT I activity, but reduced activities of G6PD, ICDH, ME and FAS, indicating that high dietary fat promoted FA oxidation and suppressed FA synthesis (Fig. 1c).

#### High-fat diet stimulated lipolytic and glycolytic pathways

**Fig. 2:**
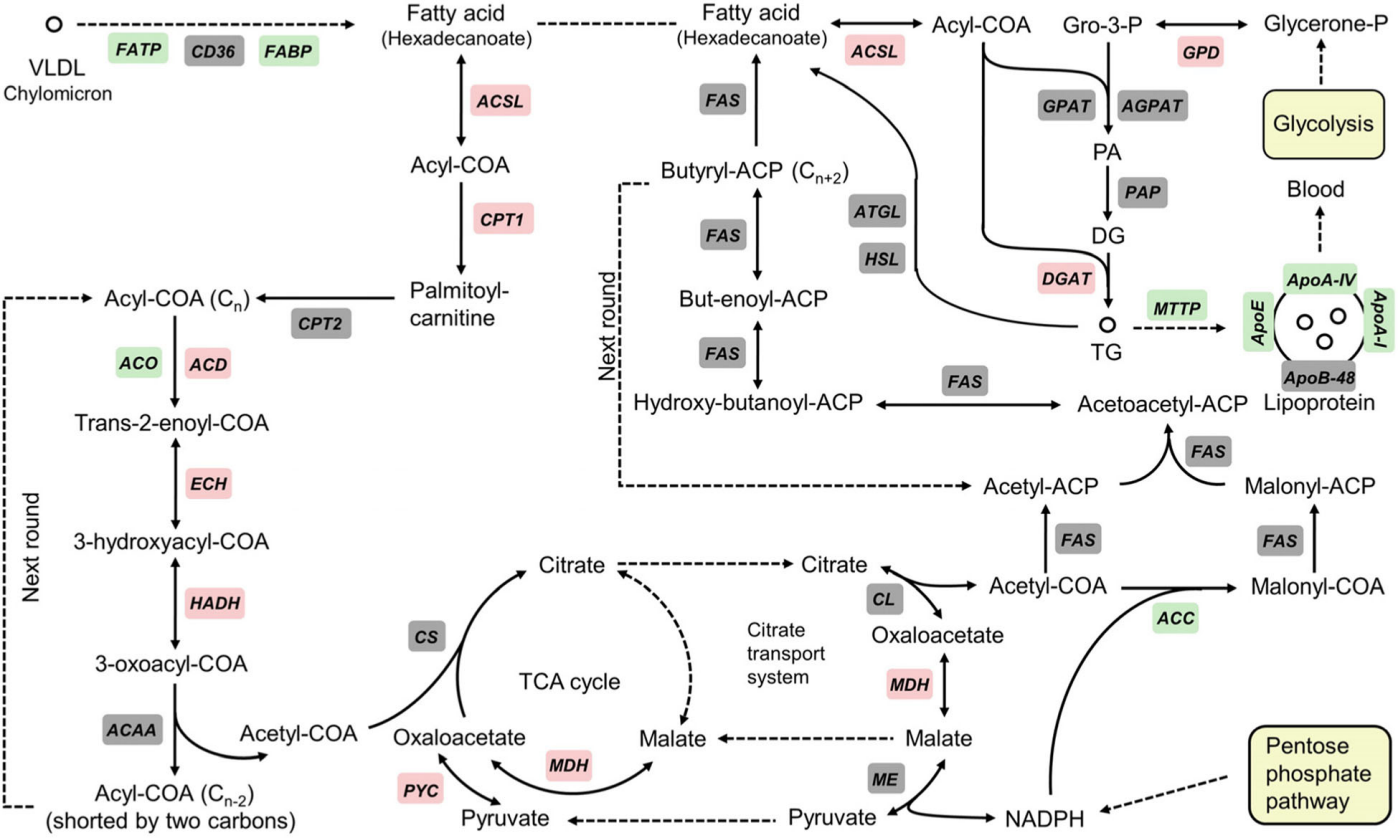
Differentially expressed genes involved in lipid metabolism. The up-regulated genes (Probability ≥0.8, and the absolute value of log_2_ (Ratio) ≥ 1) are highlighted in red. The down-regulated genes (Probability ≥0.8, and the absolute value of log_2_ (Ratio) ≥ 1) are highlighted in green. The non DEGs are highlighted in grey. PA, phosphatidic acid; DG, diacylglycerol; VLDL, very low density lipoprotein. The magnitude changes of DEGs are shown in Additional file 6: Supplemental Table S6

The RNA-seq result indicated that high-fat diet significantly enhanced lipolysis since mRNA abundances of many lipolytic genes, such as CPT I, ACD, ECH and HADH, were up-regulated (Fig. 2). ACCα, the key regulatory gene related to FA biosynthesis, was down-regulated while the expression of DGAT, a key enzyme catalyzing TG synthesis, was up-regulated. mRNA abundances of genes involved in fat absorption and lipoprotein secretion, such as FATP, FABP, MTTP, ApoA-I, ApoA-IV and ApoE, were down-regulated.

#### High-fat diet inhibited the autophagy

**Fig. 3:**
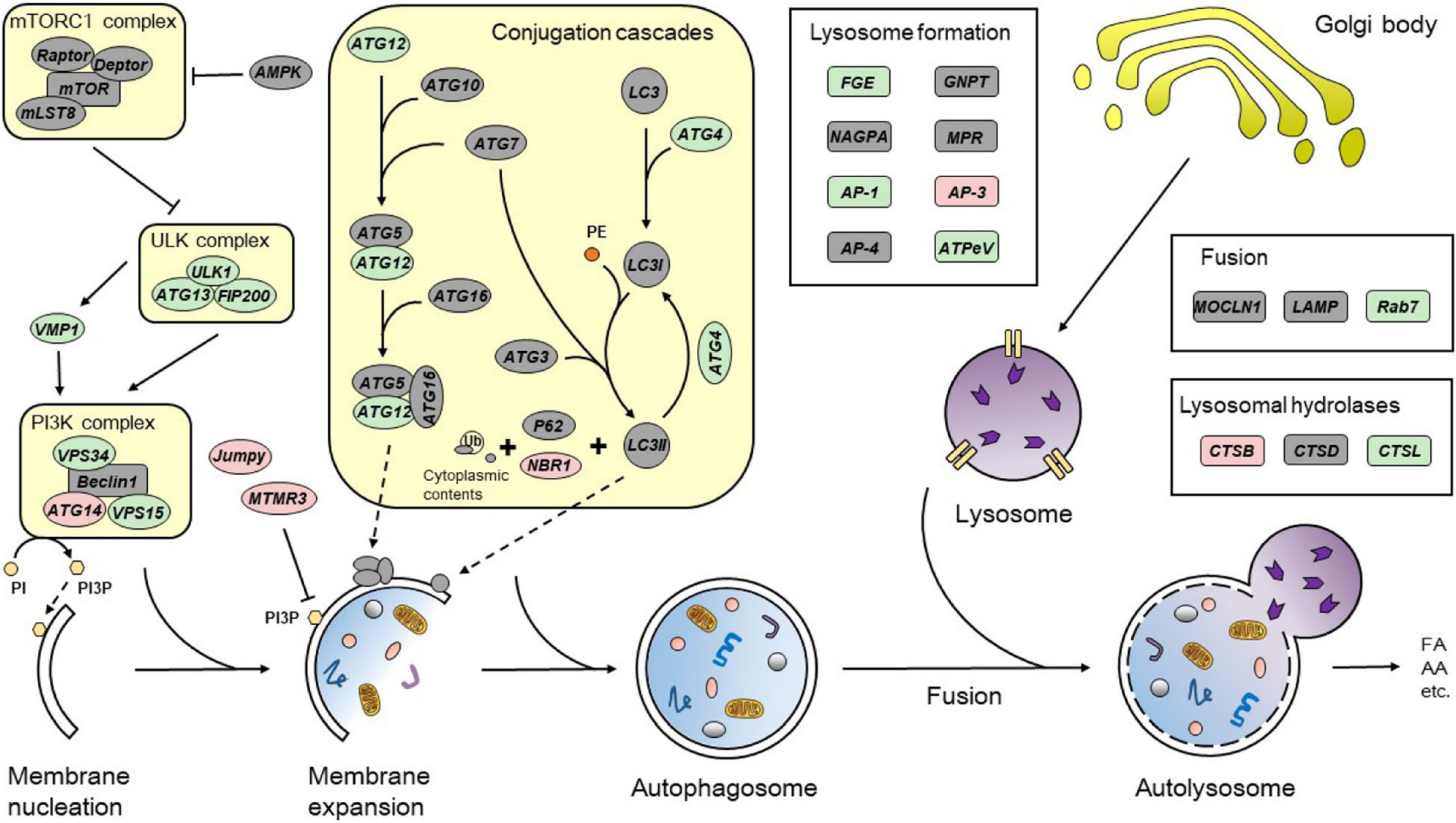
Differentially expressed genes involved in autophagy. The up-regulated genes (Probability ≥0.8, and the absolute value of log_2_ (Ratio) ≥ 1) are highlighted in red. The down-regulated genes (Probability ≥0.8, and the absolute value of log_2_ (Ratio) ≥ 1) are highlighted in green. The non DEGs are highlighted in grey. FA, fatty acid; AA, amino acid; PI, phosphatidylinositol; PE, phosphatidylethanolamine; PI3P, phosphatidylinositol-3-phosphate. The magnitude changes of DEGs are shown in Additional file 6: Supplemental Table S6

Studies pointed out that an abnormal increase in intracellular lipids impaired autophagic clearance [8]. Accordingly, we explored whether high lipid intake led to defective autophagy. Based on the pathway analysis from the DEGs of the transcriptomic sequencing, we found that high-fat diet inhibited the autophagy (Fig. 3). mRNA abundances of genes involved in the formation of autophagosome and lysosome, such as ULK1, ATG4, ATG12, ATG13, FGE and AP-1, were significantly down-regulated. The expression of Rab7, a pivotal gene that promotes the fusion of autophagosome and lysosome, was also down-regulated. However, high-fat diet did not markedly affect the expression of mTORC1 complex and AMPK. Furthermore, high lipid diet up-regulated the expression of some key genes involved in bile secretion (FXR and RXRα) and AMPK pathway (CREB1 and SREBP1), but reduced the mRNA level of autophagic gene TFEB (Table 1). Taken together, these observations confirmed that high fat diets supressed autophagy.

**Table 1:** DEGs between control and high-fat diet group from transcriptome analysis

DEGs	KEGG	Up-Down-Regulation	Log_2_(Ratio)	Pathway
FXR	K08537	Up	1.26	Bile secretion
RXRα	K08524	Up	1.29	Bile secretion
PPARα	K07294	Up	2.27	PPAR signaling pathway
CREB1	K09048	Up	3.37	AMPK signaling pathway
SREBP1	K07197	Up	2.58	AMPK signaling pathway
TFEB	K15590	Down	−1.05	Autophagy

### In vitro study

#### Activation of autophagy aggravated FA-induced lipotoxicity and apoptosis

**Fig. 4:**
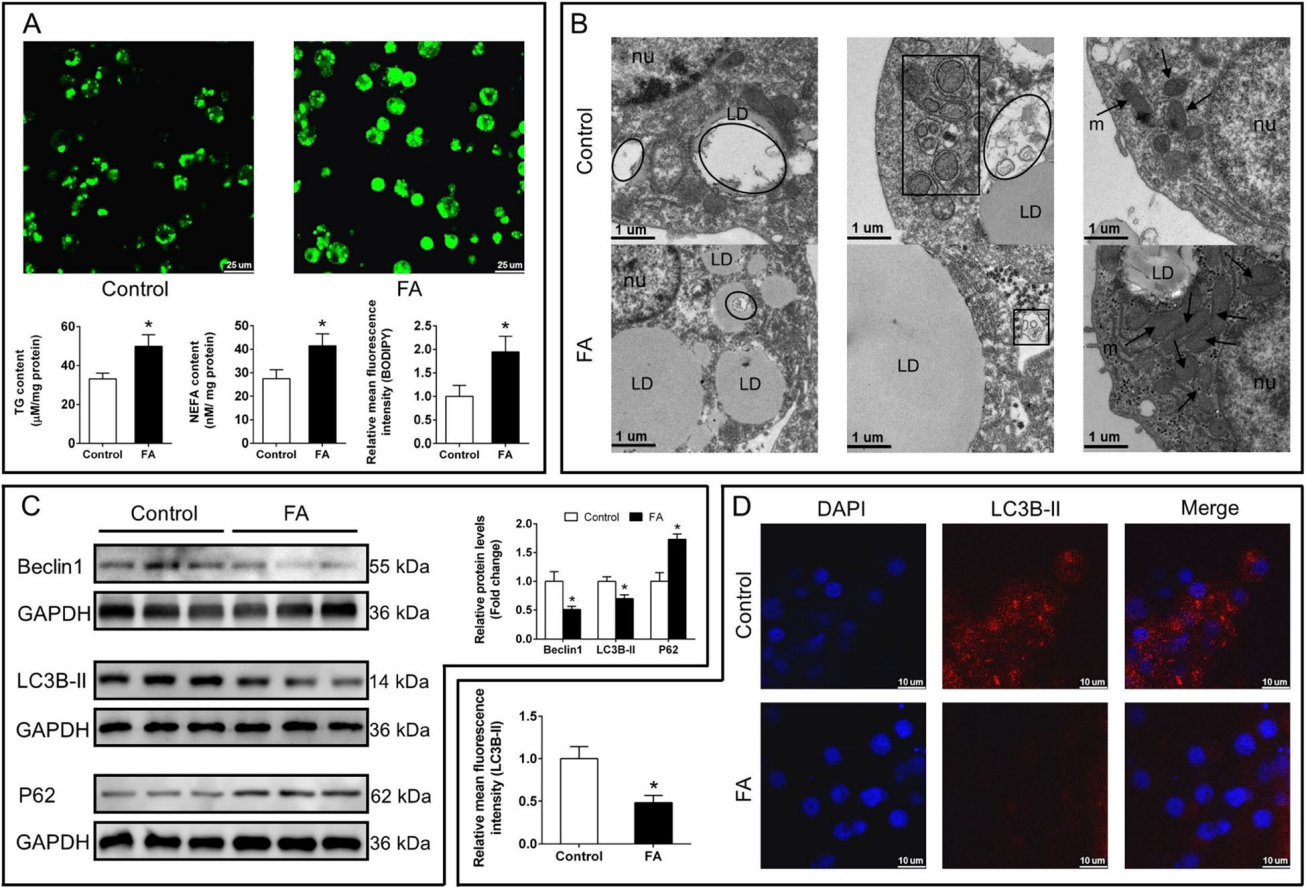
Effects of FA incubation on lipid accumulation, ultrastructures and autophagy in yellow catfish hepatocytes at 48 h. **a** The contents of TG and NEFA, and LD staining and fluorescence intensity of Bodipy. **b** Representative TEM image of hepatocytes. Nu: nucleus, LD: lipid droplet, arrowheads: mitochondria (m), black box: autophatic vesicles, oval frame: lipid clearance by autophagy. **c** Western blot analysis of Beclin1, LC3B-II and P62. **d** Immunofluorescence analysis of LC3B-II. Values are means ± SEM (*n* = 3). Asterisks (∗) indicate significant differences between the control and FA group (*p* < 0.05)

Given the evidence from the in vivo study that high fat diets induced lipid deposition and inhibited the autophagy, we first verified whether increased FA uptake directly inhibited autophagy. As expected, FA incubation significantly increased the contents of TG and NEFA, sizes and numbers of LD (Fig. 4a). By using the fluorescent dye Bodipy for neural lipids, we further confirmed that FA increased the numbers of LD (Fig. 4a). TEM studies indicated that FA treatment significantly increased both the sizes and numbers of lipid droplets in these hepatocytes. Moreover, FA treatment significantly increased the number of mitochondria gathering around LD but reduced the number of double-membrane autophagosome (Fig. 4b). Western blot and immunofluorescence analysis also indicated that FA incubation reduced the expression of Beclin 1 and LC3B-II (autophogic markers), but up-regulated p62/ SQSTM1 expression, which was related in autophagosome formation [26] (Fig. 4c and d).

**Fig. 5:**
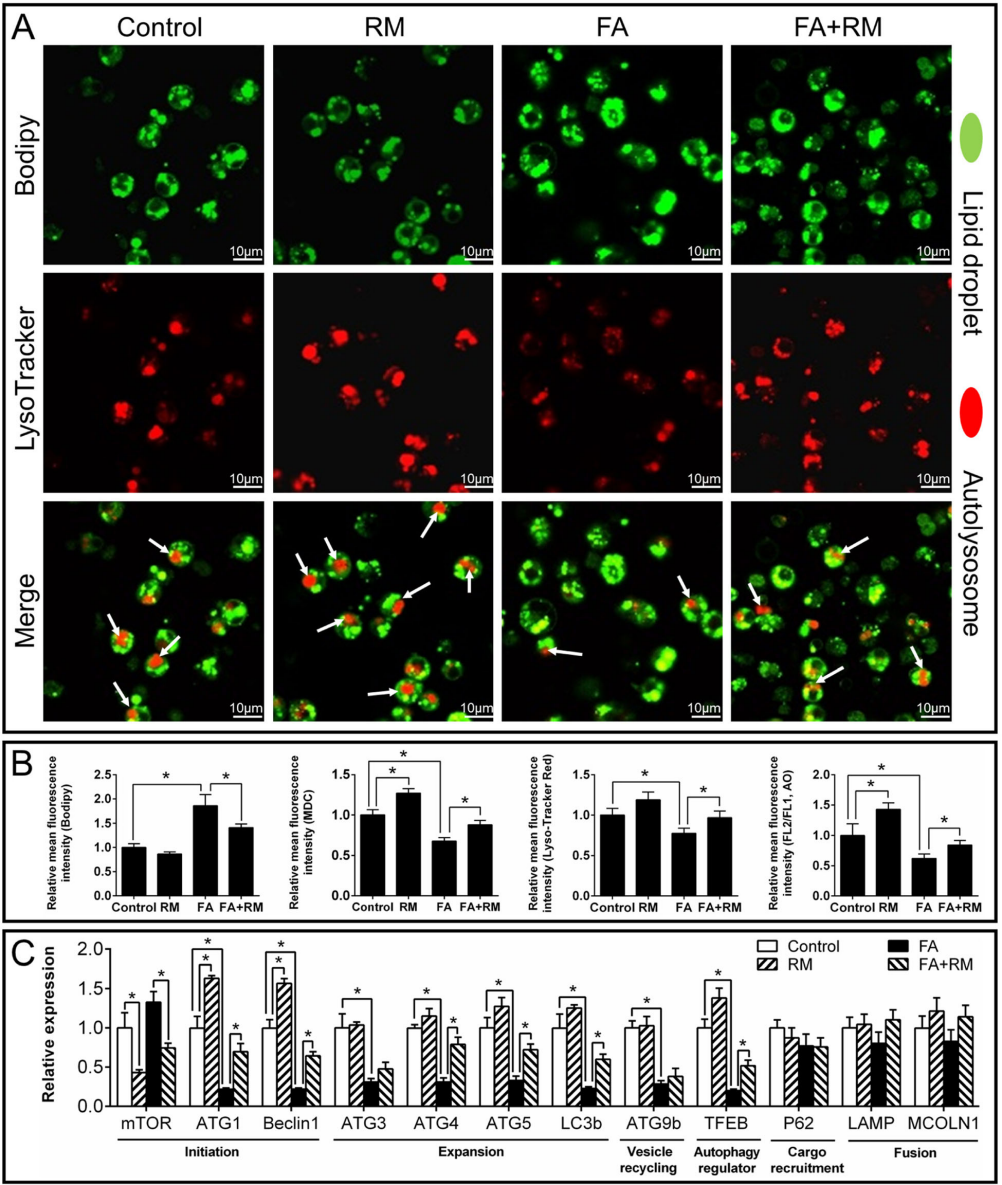
Effect of FA and rapamycin (autophagy agonist) incubation on autophagy in yellow catfish hepatocytes at 48 h. **a** Representative confocal microscopic image of hepatocytes co-stained with Bodipy and LysoTracker. **b** Relative mean fluorescence intensity of Bodipy, MDC, LysoTracker and AO staining. **c** Expression of genes involved in autophagy. RM, rapamycin. FA, oleic and palmitic acid at a ratio of 1:1. Values are means ± SEM (*n* = 3). Asterisks (∗) indicate significant differences between groups (*p* < 0.05)

**Fig. 6:**
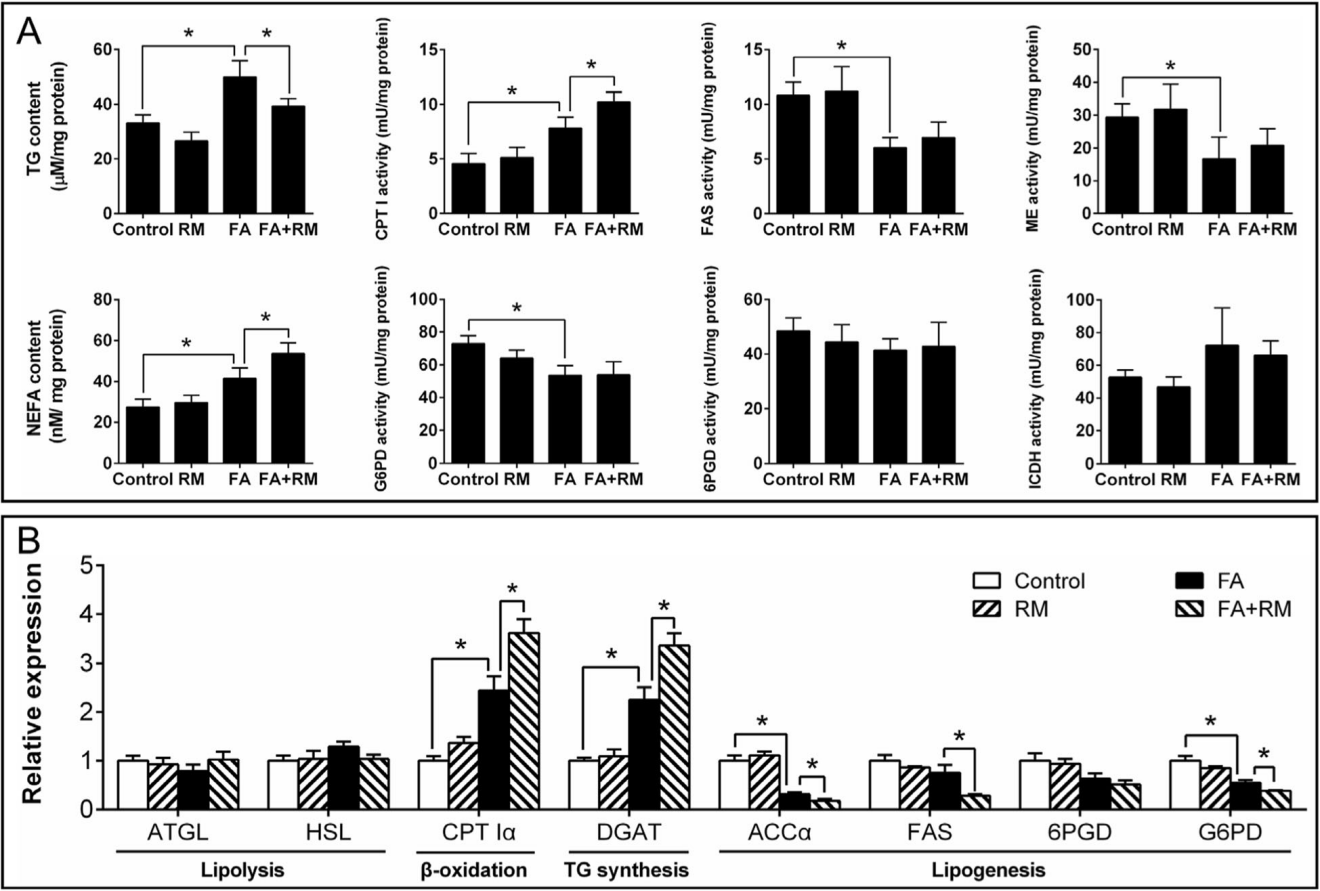
Effect of FA and RM incubation on lipid deposition and metabolism in yellow catfish hepatocytes at 48 h. **a** Contents of TG and NEFA, and enzymatic activities. **b** Expression of genes involved in lipid metabolism. RM, rapamycin. FA, oleic and palmitic acid at a ratio of 1:1. Values are means ± SEM (*n* = 3). Asterisks (∗) indicate significant differences between the two groups (*p* < 0.05)

Next, we used four methods to assess autophagosome formation. Rapamycin, an autophagy-inducing agent, acted as the positive control. The acidic intracellular vesicles were visualized by MDC, AO, and LysoTracker staining [5]. MDC is a specific marker for autophagic vesicles. Depending on the acidity of AO, autophagic lysosomes presented as orange/red fluorescent vesicles, whereas nuclei appeared green. LysoTracker Red was used to assess lysosomal activity and the function of autolysosomes. AO, MDC and LysoTracker staining demonstrated that rapamycin pretreatment markedly improved the high FA-induced inhibition of autophagy (Fig. 5a & Additional file 11: Supplemental Fig. S5). Rapamycin markedly attenuated the FA-induced down-regulation of expression of autophagy-related genes (ATG1, Beclin1, ATG4, ATG5, LC3b and TFEB) (Fig. 5c). Rapamycin pretreatment further stimulated FA-induced increase of CPT I activity, up-regulated mRNA expression of CPT Iα and DGAT, and aggravated the FA-induced reduction of the mRNA expressions of ACCα and G6PD (Fig. 6a). Rapamycin also reduced FA-induced increase of TG contents; in contrast, rapamycin pre-treatment further increased FA-induced NEFA accumulation (Fig. 6a). Interestingly, rapamycin attenuated the FA-induced inhibition of autophagy (Additional file 11: Supplemental Fig. S5A, S5C & S5D). Rapamycin increased the FA-induced increase in caspase 3 activity and up-regulated the FA-induced apoptosis rate (Additional file 12: Supplemental Fig. S6A & S6B). Taken together, all of these results indicated that activation of autophagy by rapamycin aggravated FA-induced lipotoxicity and apoptosis.

#### Inhibition of autophagy alleviated FA-induced lipotoxicity and apoptosis

To further investigate the role of autophagy in FA-induced lipotoxicity, we performed an experiment using autophagy inhibitor MA. The results showed that MA pretreatment significantly aggravated the high FA-induced inhibition of fluorescence intensity of LysoTracker and AO (Additional file 13: Supplemental Fig. S7A) and aggravated the FA-induced down-regulation of expression of autophagy-related genes (Beclin1, ATG4, ATG5 and LC3b) (Additional file 13: Supplemental Fig. S7B). MA pre-treatment promoted FA-induced increase of TG contents, but decreased FA-induced NEFA accumulation (Additional file 14: Supplemental Fig. S8A & S8B). MA pretreatment attenuated FA-induced increase of CPT I activity and mRNA expression of CPT Iα and DGAT, and alleviated the FA-induced reduction of the mRNA expressions of ACCα and G6PD (Additional file 14: Supplemental Fig. S8B & S8C). Thus, the present results suggested that inhibition of autophagy by MA alleviated FA-induced lipotoxicity.

#### FXR mediated FA-induced inhibition of autophagy and influenced lipid metabolism

**Fig. 7:**
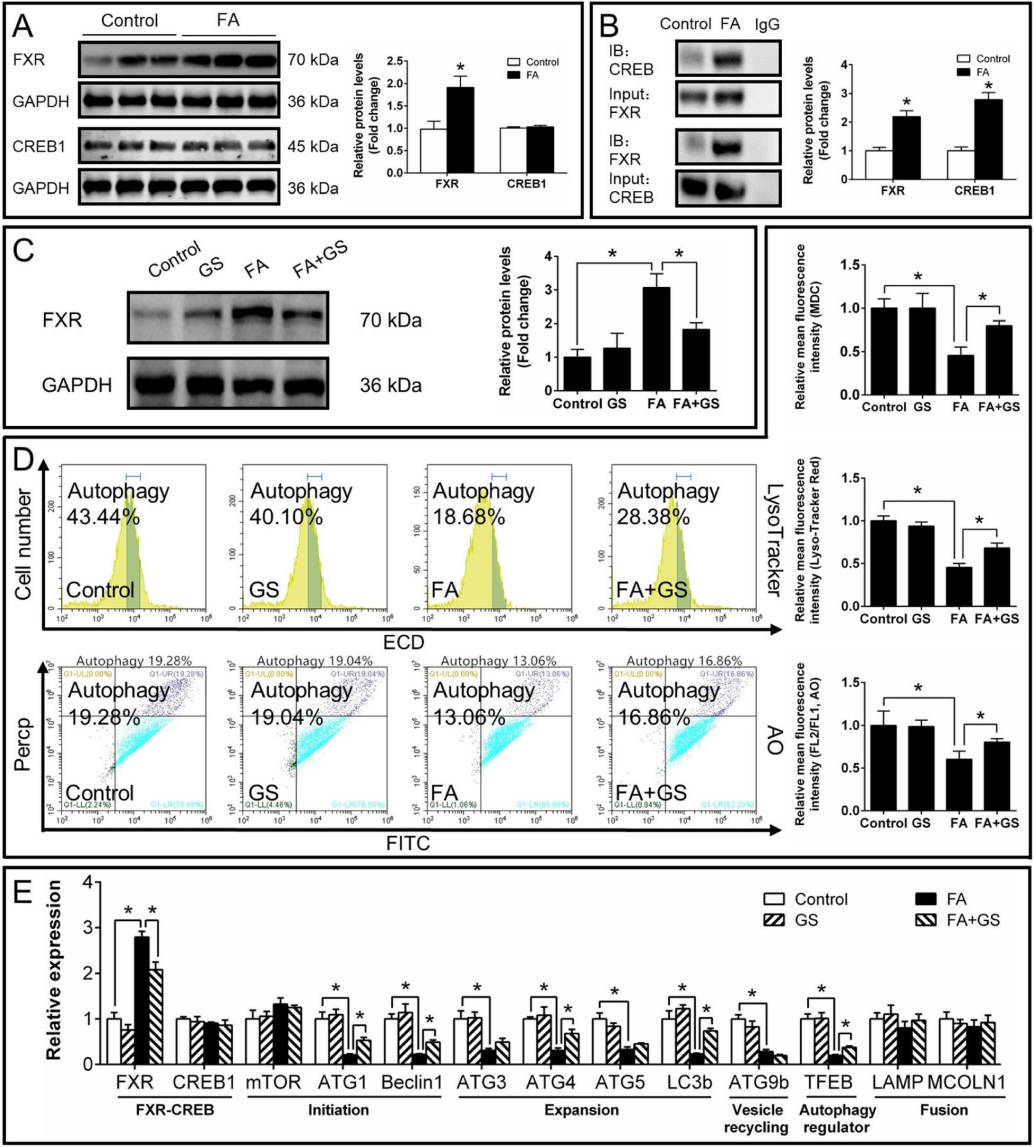
Effects of FA and guggulsterone (FXR inhibitor) incubation on FXR-CREB1 pathway and autophagy in yellow catfish hepatocytes at 48 h. **a** Protein levels of FXR and CREB1. **b** Co-IP analysis of FXR and CREB1. **c** Western blot analysis of FXR. **d** Flow cytometric analysis of LysoTracker, AO and Bodipy, and relative mean fluorescence intensity of MDC, LysoTracker, AO and Bodipy. **e** Expression of genes involved in autophagy. GS, guggulsterone. FA, oleic and palmitic acid at a ratio of 1:1. Values are means ± SEM (*n* = 3). Asterisks (∗) indicate significant differences between groups (*p* < 0.05)

**Fig. 8:**
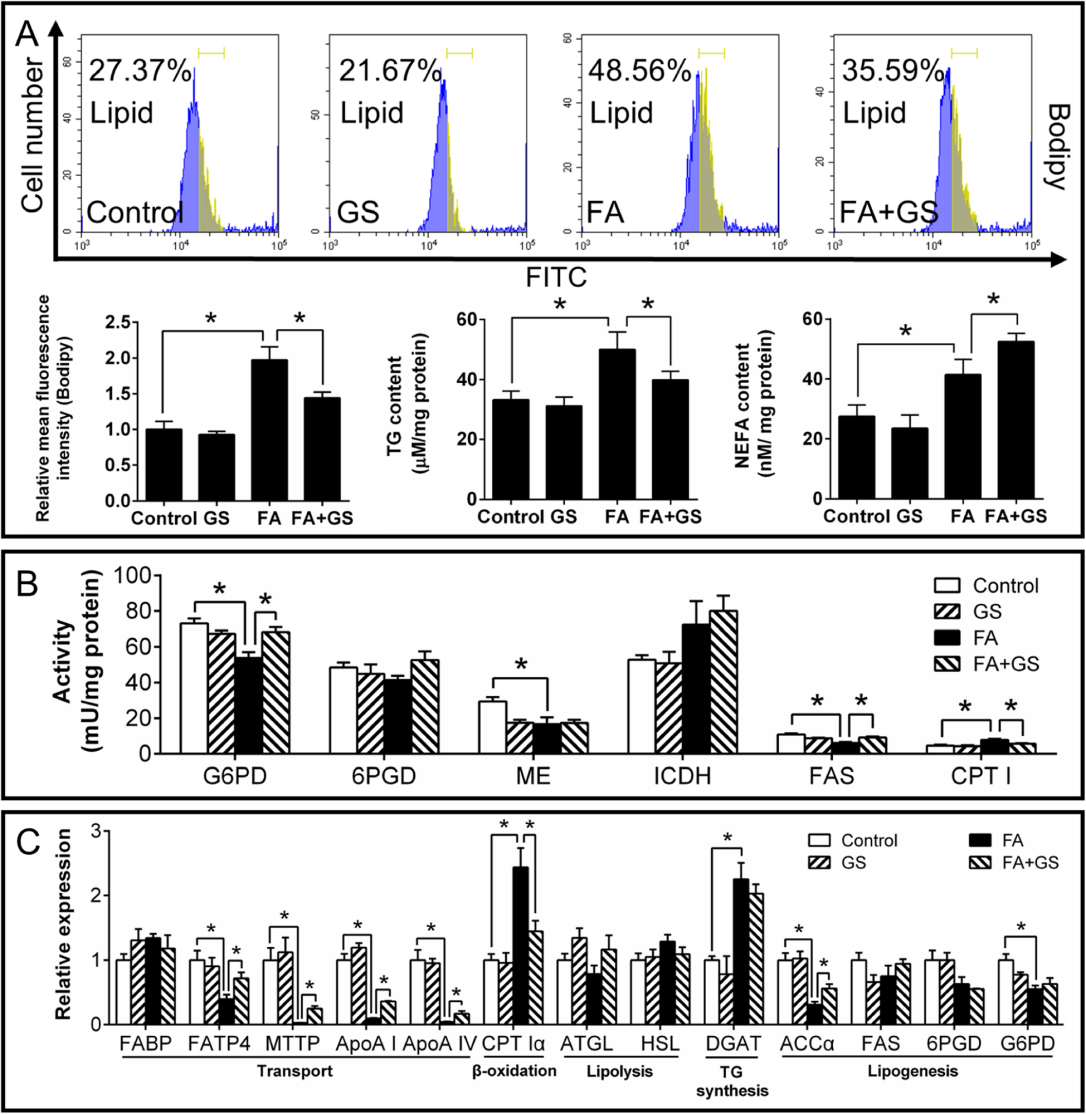
Effects of FA and guggulsterone (FXR inhibitor) incubation on lipid deposition and metabolism in yellow catfish hepatocytes at 48 h. **a** Flow cytometric analysis of Bodipy and contents of TG and NEFA. **b** Activities of enzymes involved in lipid metabolism. **c** Expression of genes involved in lipid metabolism. GS, guggulsterone. FA, oleic and palmitic acid at a ratio of 1:1. Values are means ± SEM (*n* = 3). Asterisks (∗) indicate significant differences between the two groups (*p* < 0.05)

We next investigated whether FXR, one of the key molecules regulating autophagy and lipid metabolism, would mediate the FA-induced autophagy. Western blot analysis revealed that FA up-regulated FXR protein expression (Fig. 7a) but had no effect on CREB1 protein level. IP analysis showed that FA increased the interaction between FXR and CREB (Fig. 7b). Moreover, guggulsterone (FXR inhibitor) significantly alleviated the FA-induced increase of FXR protein level (Fig. 7c) and FA-induced reduction of fluorescent intensity of MDC, LysoTracker and AO staining (Fig. 7d). Guggulsterone also significantly alleviated the FA-induced increase of FXR expression and FA-induced reduction of mRNA expression of autophagy-related genes (ATG1, Beclin1, ATG4, LC3b and TFEB) (Fig. 7e). Guggulsterone pretreatment significantly alleviated FA-induced increase of TG content, but aggravated FA-induced increase of NEFA (Fig. 8a) and apoptosis percentage (Additional file 15: Supplemental Fig. S9A & S9B). As for lipid metabolism, guggulsterone significantly alleviated the FA-induced decrease of G6PD and FAS activities, the expression of FATP4, MTP, ApoA I, ApoA IV and ACCαand suppressed the FA-induced increase of lipolytic CPT I expression and activity (Fig. 8b and c).

**Fig. 9:**
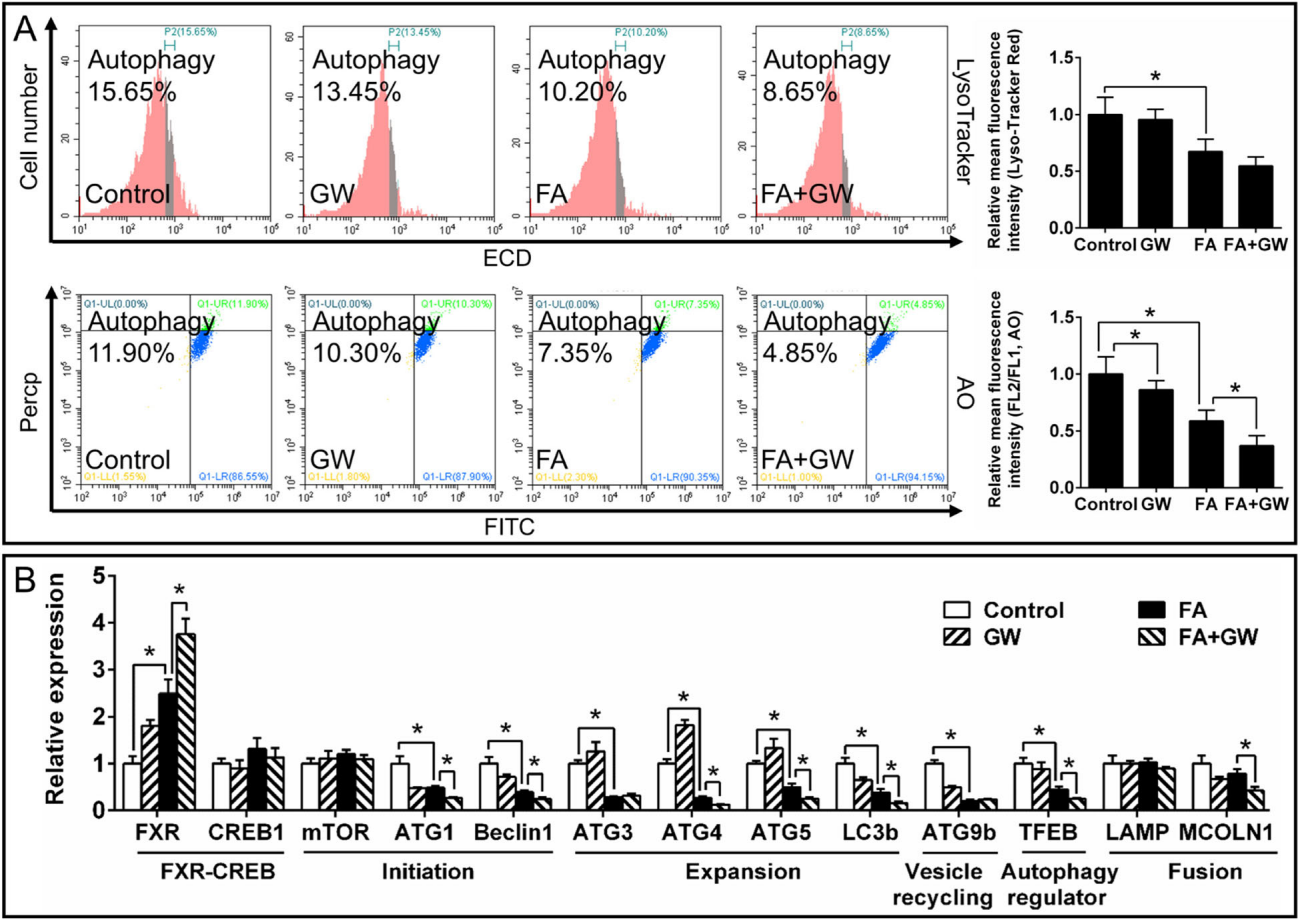
Effects of FA and GW4064 (FXR agonist) on FXR-CREB1 pathway and autophagy in yellow catfish hepatocytes at 48 h. **a** Flow cytometric analysis and relative mean fluorescence intensity of LysoTracker and AO staining. **b** Expression of genes involved in autophagy. GW, GW4064. FA, oleic and palmitic acid at a ratio of 1:1. Values are means ± SEM (*n* = 3). Asterisks (∗) indicate significant differences between the two groups (*p* < 0.05)

**Fig. 10:**
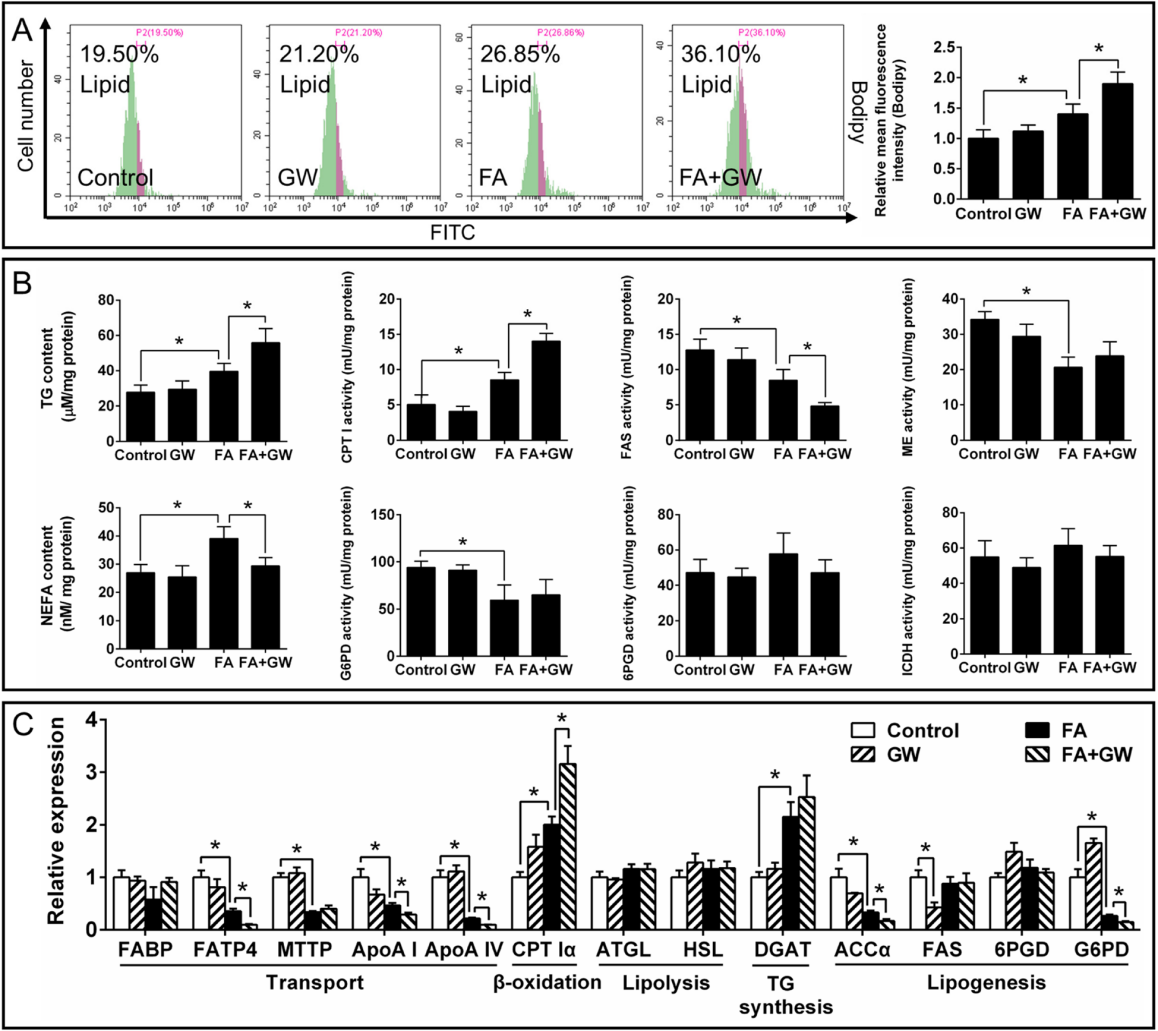
Effects of FA and GW4064 (FXR agonist) incubation on lipid deposition and metabolism in yellow catfish hepatocytes at 48 h. **a** Flow cytometric analysis of Bodipy staining. **b** Contents of TG and NEFA, and enzymatic activities. **c** Expression of genes involved in lipid metabolism. GW, GW4064. FA, oleic and palmitic acid at a ratio of 1:1. Values are means ± SEM (*n* = 3). Asterisks (∗) indicate significant differences between the two groups (*p* < 0.05)

On the other side, GW4064 (FXR agonist) pretreatment significantly aggravated the high FA-induced decrease of AO fluorescence intensity (Fig. 9a) and expression of autophagy-related genes (ATG1, Beclin1, ATG4, ATG5, LC3b and TFEB) (Fig. 9b), indicating that GW4064 markedly aggravated the FA-induced inhibition of autophagy. GW4064 also significantly aggravated FA-induced increase of TG content and Bodipy fluorescence intensity, but alleviated FA-induced increase of NEFA (Fig. 10a and b). About lipid metabolism, GW4064 pretreatment significantly aggravated the FA-induced increase of CPT I activitiy and gene expression, and aggravated FA-induced reduction of mRNA expression of FATP4, ApoA I, ApoA IV and ACCα (Fig. 10b and c). Taken together, the results indicated that FXR participated in the FA-induced inhibition of autophagy and influenced lipid metabolism by directly interacting with CREB.

## Discussion

At present, high dietary fat-induced hepatic lipotoxicity is closely related to the progression of fatty liver and has been implicated in the pathogenesis of other diseases. However, the mechanisms by which excess lipid and FA accumulation induce hepatotoxicity are not entirely clear.

The present study indicated that high fat diets induced lipid and free FA (FFA) accumulation, similar to other studies [27, 28]. The TG accumulation was once considered as the underlying reason for liver injury, but now studies suggested that the accumulation of lipid droplets played protective roles against the liptoxicity generated by FFA [27, 29, 30]. Zambo et al. [31] pointed out that FA accumulation was considered as a major cause of lipotoxicity and was more harmful to hepatocytes than TG deposition did. Taken together, our results suggest that the formation of TG may not be the cause of FA-induced lipotoxicity. Our study also indicated that high fat diets activated oxidative and ER stress, up-regulated lipolysis, reduced lipogenesis and lipid transport, and suppressed the autophagy. Similarly, other studies suggested that FA accumulation resulted in the generation of oxidative stress, and accordingly influenced the homeostasis in the ER lumen [32]. In the present study, reduced lipogenesis and lipid transport were attributable to the protective response of the liver when faced with high lipid stress. The inhibition of autophagy with excessive fat intake and high TG accumulation was also observed in many other studies [33–36]. Studies suggested that autophagy could remove excess lipid droplets and accordingly regulated lipid homeostasis, and inhibition of autophagy reduced the TG degradation, causing excessive accumulation of lipid [8, 37]. The phenomenon was also observed in the present study. Studies suggested that ER stress regulated lipogenesis and influenced lipid deposition in liver [38, 39]. Therefore, as detected in the present study, ER stress induced by impaired autophagy may further accelerate hepatic lipid accumulation. Our further investigation indicated that inhibition of autophagy contributed to alleviate lipotoxicity, and autophagy agonist rapamycin aggravated the FA-induced changes of NEFA content, expression of CPT Iα, DGAT, ACCα and G6PD, and CPT I activity (Fig. 6). On the other hand, autophagy inhibitor MA alleviated the FA-induced changes of NEFA content, expression of CPT Iα, DGAT, ACCα and G6PD, and CPT I activity (Additional file 14: Supplemental Fig. S8). Lipotoxicity is the predominant contributor to the progression of diseases like NAFLD [37]. Autophagy had the potential to influence β-oxidation by providing FFA from LD breakdown [40]. Therefore, rapamycin-induced activation of autophagy promoted FFA generation which in turn induced the elevation of β-oxidation and the inhibition of FA de novo synthesis. Given the close correlation between FFA and autophagy, inhibition of autophagy in the presence of excessive FA presumably protected cells from lipotoxicity by decreasing cellular FFA levels from TG breakdown. Taken together, inhibition of lipophagy not only helped TG deposition but also reduced FFA accumulation.

mTOR is an evolutionarily conserved protein kinase that plays a pivotal role in autophagy, and its activation leads to inhibition of autophagy [41]. However, in the present study, high fat diet had no significant influence on mTOR expression in both in vivo and in vitro studies, suggesting that high fat-induced autophagy was mTOR-independent. In agreement with these notions, several studies showed that FA induced autophagy independent of mTOR activation [27, 42].

Our next question is what triggers the fat-induced inhibition of autophagy independent of mTOR and stress pathways? FXR acts as a crucial repressor of autophagy under the fed status [11], and plays the central roles in maintaining BA homeostasis [43]. Studies suggested that FXR was activated by increased BA levels after feeding [44]. In the present study, high fat increased bile acid content, and mRNA and protein expression of FXR in the liver, indicating high fat-induced activation of FXR, similar to the studies in mice [45]. Thus, we further tested whether FXR regulated FA-induced inhibition of autophagy. The present study indicated that guggulsterone-mediated FXR inhibition significantly alleviated the FA-induced reduction of fluorescent intensity of MDC, LysoTracker and AO staining (Fig. 7d). Guggulsterone also significantly alleviated FA-induced reduction of mRNA expression of autophagy-related genes (ATG1, Beclin1, ATG4, LC3b and TFEB). On the other hand, GW4064-mediated FXR activation markedly aggravated the FA-induced inhibition of autophagy (Fig. 9). The present results confirmed that FXR participated in the FA-induced inhibition of autophagy. Similarly, other studies indicated the close association of FXR and autophagy in the liver of animal models under the fed conditions [10, 11]. The down-regulation of FXR expression increased lipophagy in several non-fasted cell lines [11]. Seok et al. [11] pointed out that FXR-mediated inhibition of autophagy was independent of mTOR activity. Moreover, our IP analysis indicated that FA increased the interaction between FXR and CREB (Fig. 7b). FA-induced inhibition of autophagy was partly recovered by FXR inhibition (Fig. 7), indicating that FXR-CREB axis mediated inhibition of autophagy. Similarly, Seok et al. [11] reported that the activation of FXR interacted with CREB, and the transcriptional complex FXR-CREB inhibited autophagy. Guggulsterone also alleviated the FA-induced increase of TG content, CPT Iα mRNA expression and CPT I activity, and reversed the FA-induced decrease of G6PD and FAS activities, mRNA levels of FATP4, MTP, ApoA I, ApoA IV and ACCα (Fig. 8), thereby impacting lipid metabolism. In contrast, GW4064 aggravated the FA-induced increase of TG content, CPT Iα mRNA expression and CPT I activity, and suppressed the FA-induced decrease of FAS activity, mRNA levels of FATP4, ApoA I, ApoA IV, ACCα and G6PD (Fig. 10). As a matter of fact, FXR regulated lipid metabolism in various ways. For example, FXR regulated TG secretion and synthesis, FA absorption and oxidation [46]. In addition, guggulsterone aggravated FA-induced increase of NEFA and apoptosis percentage (Fig. 8, Additional file 15: Supplemental Fig. S9), while GW 4064 alleviated FA-induced increase of NEFA (Fig. 10). These results indicated that the inhibition of FXR aggravated the lipotoxicity. Taken together, FXR signals constituted a pivotal link between autophagy inhibition and high fat intake; FXR activation was necessary to prevent the FA-induced lipotoxicity. Thus, our study revealed a novel mechanism for autophagy activation, which suggested the potential and new therapeutic methods for curing fatty liver.

## Conclusion

High fat-induced inhibition of autophagy played key roles in lipid deposition, which counteracted hepatic lipotoxicity through suppressing FA generation from TG/LD breakdown. In this process, FXR-CREB was a key physiological switch regulating autophagy and lipid metabolism, and represented a cellular defense against liver toxicity response to lipid overload. The nutrient-sensing FXR-CREB axis, which closely modulates the autophagy network, may emerge as potential and new molecular targets for curing diseases involved in autophagy dysfunction and metabolic disorders, including NAFLD.

## Supplementary information

**Additional file 1: Supplemental Table S1.** Feed formulation and proximate analysis of experimental diets.

**Additional file 2: Supplemental Table S2.** Summary of output statistics by sequencing.

**Additional file 3: Supplemental Table S3.** Statistics of assembly quality.

**Additional file 4: Supplemental Table S4.** Summary of annotation.

**Additional file 5: Supplemental Table S5.** Primers used for Q-PCR analysis.

**Additional file 6: Supplemental Table S6.** The magnitude changes of DEGs.

**Additional file 7: Supplemental Fig. S1.** Scatter plots showing the correlation between the gene expression profiles of adequate-fat (AF, control) and high-fat (HF, treatment) groups. X-axis and Y -axis mean log2 value of gene expression. Differentially expressed genes are indicated in red (up-regulated expression) and blue (down-regulated expression). Brown means genes that were not differentially expressed.

**Additional file 8: Supplemental Fig. S2.** GO functional classification of DEGs. X axis means number of DEGs (the number is presented by its square root value). Y axis represents GO terms. All GO terms are grouped in to three ontologies: red is for biological process, blue is for cellular component and green is for molecular function.

**Additional file 9: Supplemental Fig. S3.** KEGG functional classification of DEGs. X axis means number of DEGs. Y axis represents the second KEGG pathway terms, and then the second pathway terms are grouped in the top pathway terms as indicated in different color.

**Additional file 10: Supplemental Fig. S4.** Comparison of mRNA levels between RNA-seq and Q-PCR results. The y-axis is the gene expressed fold change and the x-axis is the gene name. The correlation coefficient between RNA-seq and Q-PCR results was 0.903 (*p* < 0.001).

**Additional file 11: Supplemental Fig. S5.** Effects of FA and rapamycin (autophagy agonist) incubation on autophagy in yellow catfish hepatocytes at 48 h. A) Representative confocal microscopic image of hepatocytes co-stained with MDC and LysoTracker. B) Representative confocal microscopic image of hepatocytes stained with AO. C) Flow cytometric analysis of Bodipy, LysoTracker and AO staining. RM, rapamycin. FA, oleic and palmitic acid at a ratio of 1:1. Values are means ± SEM (*n* = 3). Asterisks (∗) indicate significant differences between the two groups (*p* < 0.05).

**Additional file 12: Supplemental Fig. S6.** Effects of FA and RM on apoptosis in yellow catfish hepatocytes. A) Flow cytometric analysis of apoptosis. B) caspase 3 activity, cell viability and quantitation of relative mean fluorescence intensity of apoptosis. RM, rapamycin. FA, oleic and palmitic acid at a ratio of 1:1 Values are means ± SEM (*n* = 3). Asterisks (∗) indicate significant differences between the two groups (*p* < 0.05).

**Additional file 13: Supplemental Fig. S7.** Effects of FA and 3-methyladenine (autophagy inhibitor) on autophagy in yellow catfish hepatocytes at 48 h. A) Flow cytometric analysis and relative mean fluorescence intensity of LysoTracker and AO staining. B) Expression of genes involved in autophagy. MA, 3-methyladenine. FA, oleic and palmitic acid at a ratio of 1: 1. Values are means ± SEM (*n* = 3). Asterisks (∗) indicate significant differences between the two groups (*p* < 0.05).

**Additional file 14: Supplemental Fig. S8.** Effects of FA and 3-methyladenine (autophagy inhibitor) incubation on lipid deposition and metabolism in yellow catfish hepatocytes at 48 h. A) Flow cytometric analysis of Bodipy staining. B) Contents of TG and NEFA, and enzymatic activities. C) Expression of genes involved in lipid metabolism. MA, 3-Methyladenine. FA, oleic and palmitic acid at a ratio of 1:1. Values are means ± SEM (*n* = 3). Asterisks (∗) indicate significant differences between the two groups (*p* < 0.05).

**Additional file 15: Supplemental Fig. S9.** Effect of FA and guggulsterone (FXR inhibitor) incubation on apoptosis in yellow catfish hepatocytes at 48 h. A) Flow cytometric analysis of apoptosis. B) caspase 3 activity, cell viability and quantitation of relative mean fluorescence intensity of apoptosis. GS, guggulsterone. FA, oleic and palmitic acid at a ratio of 1:1. Values are means ± SEM (*n* = 3). Asterisks (∗) indicate significant differences between the two groups (*p* < 0.05).

## Data Availability

The datasets used and/or analysed during the current study are available from the corresponding author on request.

## Abbreviations

6PGD: 6-phosphogluconate dehydrogenase
ACC: Acetyl-CoA carboxylase
ATG: Autophagy-related protein
Apo: Apolipoprotein
ATGL: Adipose triglyceride lipase
B2M: Beta-2 microglobulin
CPT I: Carnitine palmitoyl transferases I
CREB1: Cyclic AMP-responsive element-binding protein 1
DGAT: Diacylglycerol acyltransferase
eIF2α: eukaryotic translation initiation factor 2 subunit 1
ELFA: Elongation factor aipha
FABP: Fatty acid binding protein
FAS: Fatty acid synthase
FATP: Fatty acid transport protein
FUBP: Far upstream element-binding protein
FXR: Farnesoid X receptor
G6PD: Glucose-6-phosphate dehydrogenase.
GAPDH: Glyceraldehyde-3-phosphate dehydrogenase
GPAT: Glycerol-3-phosphate- acyltransferase
HPRT: Hypoxanthine-guanine phosphoribosyl transferase
HSL: Hormone sensitive lipase
ICDH: Isocitrate dehydrogenase
LAMP: Lysosomal-associated membrane protein
LC3: Autophagy-related protein 8
MCOLN1: Mucolipin 1
ME: Malic enzyme
P62: Sequestosome 1
PPAR: Peroxisome proliferators-activated receptor
RXRα: Retinoid X receptor alpha
SREBP 1: Sterol-regulator element-binding protein 1
TFEB: Transcription factor EB
TUBA: Tubulin alpha chain
ULK1: Serine/threonine-protein kinase ULK1
