# Supplementary material for: FXR-mediated inhibition of autophagy contributes to FA-induced TG accumulation and accordingly reduces FA-induced lipotoxicity
Source: Cell Commun Signal. 2020 Mar 20;18:47. doi: 10.1186/s12964-020-0525-1 (PMC7082988; doi:10.1186/s12964-020-0525-1)
Supplement: Supplementary file 2 — Additional file 1: Supplemental Table S1. Feed formulation and proximate analysis of experimental diets. [file 12964_2020_525_MOESM1_ESM.doc]

**Supplemental Table S1 Feed formulation and proximate analysis of experimental diets**

|  | Adequate fat diet | High fat diet |
| --- | --- | --- |
| Ingredients (g kg -1 ) |  |  |
| Casein | 260 | 260 |
| White fish meal | 250 | 250 |
| Gelatin | 20 | 20 |
| Fish oil | 30 | 50 |
| Soybean oil | 30 | 50 |
| Starch | 200 | 200 |
| Ascorbyl-2-polyphosphate | 10 | 10 |
| NaCl | 10 | 10 |
| CaH_2_PO_4_ ·2H_2_O | 10 | 10 |
| Vitamin mix | 5 | 5 |
| Mineral mix | 5 | 5 |
| Betaine | 10 | 10 |
| Cellulose | 160 | 120 |
| Proximate analysis (percentage of dry matter basis) | | |
| Moisture | 6.92 | 8.58 |
| Crude ash | 6.04 | 5.94 |
| Crude protein | 40.91 | 39.72 |
| Crude lipid | 11.34 | 15.41 |
