# Supplementary material for: FXR-mediated inhibition of autophagy contributes to FA-induced TG accumulation and accordingly reduces FA-induced lipotoxicity
Source: Cell Commun Signal. 2020 Mar 20;18:47. doi: 10.1186/s12964-020-0525-1 (PMC7082988; doi:10.1186/s12964-020-0525-1)
Supplement: Supplementary file 3 — Additional file 2: Supplemental Table S2. Summary of output statistics by sequencing. [file 12964_2020_525_MOESM2_ESM.doc]

**Supplemental Table S2 Summary of output statistics by sequencing**

| Samples | Total raw reads (Mb) | Total clean reads (Mb) | Total clean Bases (Gb) | Clean Reads Q20 (%) |
| --- | --- | --- | --- | --- |
| AF 1 | 82.10 | 66.17 | 6.62 | 96.87 |
| AF 2 | 84.62 | 66.56 | 6.66 | 96.73 |
| AF 3 | 84.60 | 66.77 | 6.68 | 96.58 |
| HF 1 | 84.61 | 66.17 | 6.62 | 96.65 |
| HF 2 | 82.14 | 65.34 | 6.53 | 96.84 |
| HF 3 | 84.62 | 66.72 | 6.67 | 96.71 |

AF1, AF 2, AF 3 represent 3 biological replicates of adequate-fat (control) group; HF 1, HF 2, HF 3 represent 3 biological replicates of high-fat diet group; The base quality score of 20 (Q20) means an error probability of 1%, based on Phil Green’s PHRED base-calling software.
