# Supplementary material for: FXR-mediated inhibition of autophagy contributes to FA-induced TG accumulation and accordingly reduces FA-induced lipotoxicity
Source: Cell Commun Signal. 2020 Mar 20;18:47. doi: 10.1186/s12964-020-0525-1 (PMC7082988; doi:10.1186/s12964-020-0525-1)
Supplement: Supplementary file 4 — Additional file 3: Supplemental Table S3. Statistics of assembly quality. [file 12964_2020_525_MOESM3_ESM.doc]

**Supplemental Table S3 Statistics of assembly quality**

| Samples | Total number | Total length (bp) | Mean length (bp) | N50 | GC (%) |
| --- | --- | --- | --- | --- | --- |
| AF1 | 48,103 | 44,686,177 | 928 | 1705 | 44.39 |
| AF2 | 54,763 | 53,544,622 | 977 | 1823 | 44.28 |
| AF3 | 49,979 | 47,151,834 | 943 | 1732 | 44.36 |
| HF1 | 56,850 | 55,143,832 | 969 | 1780 | 44.31 |
| HF2 | 49,704 | 48,141,527 | 968 | 1785 | 44.60 |
| HF3 | 56,826 | 56,166,682 | 988 | 1862 | 44.39 |
| All-Unigene | 69,307 | 89,105,992 | 1285 | 2492 | 44.10 |

N50 indicates the median length of all non-redundant sequences, and the higher N50 value represents the better the quality of assembly; GC percentage is proportion of guanidine and cytosine nucleotides among total nucleotides.
