# Supplementary material for: FXR-mediated inhibition of autophagy contributes to FA-induced TG accumulation and accordingly reduces FA-induced lipotoxicity
Source: Cell Commun Signal. 2020 Mar 20;18:47. doi: 10.1186/s12964-020-0525-1 (PMC7082988; doi:10.1186/s12964-020-0525-1)
Supplement: Supplementary file 5 — Additional file 4: Supplemental Table S4. Summary of annotation. [file 12964_2020_525_MOESM4_ESM.doc]

**Supplemental Table S4** Summary of annotation

|  | Number of unigene hits | Percentage |
| --- | --- | --- |
| All-unigenes | 69,307 |  |
| All annotated unigenes | 49,386 | 71.26% |
| Annotated to NR database | 40,398 | 58.29% |
| Annotated to NT database | 40,633 | 58.63% |
| Annotated to Swiss-Prot database | 34,044 | 49.12% |
| Annotated to KEGG database | 33,830 | 48.81% |
| Annotated to KOG database | 30,157 | 43.51% |
| Annotated to Interpro database | 30,536 | 44.06% |
| Annotated to GO database | 11,745 | 16.95% |
