# Supplementary material for: FXR-mediated inhibition of autophagy contributes to FA-induced TG accumulation and accordingly reduces FA-induced lipotoxicity
Source: Cell Commun Signal. 2020 Mar 20;18:47. doi: 10.1186/s12964-020-0525-1 (PMC7082988; doi:10.1186/s12964-020-0525-1)
Supplement: Supplementary file 6 — Additional file 5: Supplemental Table S5. Primers used for Q-PCR analysis. [file 12964_2020_525_MOESM5_ESM.doc]

**Supplemental Table S5 Primers used for Q-PCR analysis**

| Gene | Forward primer (5'-3') | Reverse primer (5'-3') |
| --- | --- | --- |
| 6PGD | CTGCTGCTGGACTCCTTCTT | GTTGTGTCTGTAACCGTCGTAA |
| β-actin | GTGCGTGACATCAAGGAGAAG | CGAGGAAGGATGGCTGGAA |
| ACCα | ACTTCTGCTGTGGTTGTCCTAT | GCATCCATCGTGGGTCATA |
| ApoA I | CCTGGATGGAGGCGAATATG | CTGGTTGGCAAAGGGTTTCAG |
| ApoA IV | CATTGAGCAAGACGGAAACA | CAAAGGGTTTCAGTGGGGTG |
| ATF6 | CAGTAAGAAGGCGGAAGTG | TGGTGAGGGGCGTAGTAGAC |
| ATG1 | GGAGGGTGTTGTGATGTTTGAGGC | CTTGCTGGTGTAGGCAGGTTGTG |
| ATG3 | GGAAGGCTGAGGCAAGCGGAGA | TGGGTGGTTTTCAATGGTGACTGTTTT |
| ATG4 | ATGGAGGCAGTTTTAGCCAAGTAT | TGTATGTAAACCACAGCCGTGAA |
| ATG5 | CAGAACCGTTTTATCTTCTCCTACCG | CGTCTACATCTTCAGCTTTCACGACTT |
| ATG9b | ACCAGCAGGCAGAAAACGGCAAGA | GTATCGGGAGCGGTGGCGGAGTC |
| ATGL | ATCTGGTGAAGGTGCTGAAGT | CTCTGACTGTGGCAGGTTGT |
| B2M | GCTGATCTGCCATGTGAGTG | TGTCTGACACTGCAGCTGTA |
| Beclin1 | CTCAACTGGACCGCCTGAAGAA | CACTCCACAGGAACGCTGGGTA |
| BLOC1S5 | GCTCCCTCTCCTGGATTCTC | GGCTTTAGGGAGTGTCGTCT |
| CD22 | TCTCACATTCTGGGGTCCAC | CTCACAAGGGGCCAAAATCC |
| CNKSR2 | TGAGTTGGTGCCGGTCTTAC | TGAGTTGGTGCCGGTCTTAC |
| CPT Iα | CGCTCCTGCTCCAATGAGA | GAGACCACATAGAGGCAGAAGA |
| CREB1 | GCTCAGCCGTCCGTCATC | TCCCTTCTTTTCTGGGTGTC |
| CRT | CGTGAAGAAGAGGACAAGA | AGAATAGGAGGCATAGCAG |
| CYP24A1 | GCCGATGCCAAAAGGTACAT | CTTTCACCCTCCATCCCCTT |
| DGAT | ACATCTTCGGTTATCATCCTCAC | ACGATGACCACAGCGTTTCC |
| DIABLO | GGTGTGTGCTTGTGTTCAGT | TTGAAGAGCGATGAGGGTGT |
| DLG2 | ACCCAGGTCCAGTCTCAATG | TTGACGGGCATAGCTCCTAG |
| eIF2α | AGGATGTGGTGATGGTGAA | CGATGCGGATAAGTTTGTT |
| ELFA | GTCTGGAGATGCTGCCATTG | AGCCTTCTTCTCAACGCTCT |
| EP400 | TCGTGTATCCTTCCTACCGC | GGATCGACAATGCCTCCAAC |
| FABP | TCGTGGTGGTGAGGACTTTG | CATTGAGCAAGACGGAAACA |
| FAS | CATCATCACTGGAGGTCTTGGA | TACGAATGCCTGATCTGGAAGT |
| FATP4 | TGCCCCTCACATAGTTGCTG | CACTTCCTCGAACATCCCTCAT |
| FUBP | GCAGGATTACACCAAAGCGT | CTTCACATTACTGGCCCTGC |
| FXR | ATGCTTGCGGAATGTTTACT | TTGTGGATGTGACCTGCTTAG |
| G6PD | GAGAAGCCTGCCTCAACCA | GGATCGTCCAAGTAGCCAAGT |
| GAPDH | AAAGTCATCCCCGAGCTCAA | CTTCAGACGCAGCCTTCATC |
| GRP78 | GCTCCACTCGTATCCCCAA | TCCGTAAGCCACAGCCTCA |
| GRP94 | GCCAAGGAGAAGAGAGAGG | ACCAGAGCACAGGGAGATT |
| HPRT | ATGCTTCTGACCTGGAACGT | TTGCGGTTCAGTGCTTTGAT |
| HSL | ACCATTGCTCCACCGTCTG | CGTCTCACTATCCTGTCCTTCA |
| IRE1α | CCTACTTCACATCCCGCTT | AGTTCGCTTGACTTTGCTC |
| LAMP | CAGCAACAGCAGTAGTGGGA | AGCTCAGTGTAAGGTTGGCC |
| LC3b | CCTGACCACGTCAACATGAGCGAACT | GGAAATGGCGGCAGACACGGAGA |
| MCOLN1 | ATCGTGACCCAAACTCGCTT | GGTCGGCGGGTATATCTTGG |
| mTOR | TGTGCCGACCATTTTCTG | GATCTGGATCTGTGATGCCTA |
| MTTP | CAGTCGCTCAATCTGCTCAC | ATCACTCCCACAGACACCAG |
| NBR1 | CAGGGGTTTATGGATGCAGC | AAATCCGGAAGCCTGACAGA |
| P62 | TTTCTCAAACCTCCAAAATGTCGC | GGGAAGTCACGCTTGTGCTCCTT |
| PERK | GAAAAATAACATGGTGCCTCGG | GCCGAGGCACCATGTTATTTTTC |
| RASGRP3 | CACTCTGTTCCTTCCCTCGT | GGATCTCAGTGCGCTTTCAG |
| RPL7 | GCGCCAGATCTTCAATGGAG | CTCATTCTGCCATGACCACG |
| SLC19A1 | CAAGCACGTGGAAAGCAACA | CAGGCCAGCTGTGTATGTCA |
| TBP | AGTCCCATGATGCCCTATGG | GCAACAGCTTGGGAATGGAA |
| TFEB | ACCAGCGACCTCCTCCTAAT | AGCTCAAATCTCCCAGGCAC |
| TRIM16 | GAGATCACCCTGGACCCAAA | CCGCGACCTTTTCTGTTGAT |
| TUBA | CACTTCCCTCTTGCCACCTA | ACGGTACAGGAGACAACAGG |
| XBP1 | GTGCTTCTCATTTCTTCATC | ACTCTGTTCTTCAGTTTCC |
