# Supplementary material for: FXR-mediated inhibition of autophagy contributes to FA-induced TG accumulation and accordingly reduces FA-induced lipotoxicity
Source: Cell Commun Signal. 2020 Mar 20;18:47. doi: 10.1186/s12964-020-0525-1 (PMC7082988; doi:10.1186/s12964-020-0525-1)
Supplement: Supplementary file 7 — Additional file 6: Supplemental Table S6. The magnitude changes of DEGs. [file 12964_2020_525_MOESM6_ESM.doc]

**Supplemental Table S6 The magnitude changes of DEGs**

| DEGs | KEGG | Up-Down-Regulation | Log_2_(Ratio) | Pathway |
| --- | --- | --- | --- | --- |
| ACC | K11262 | Down | -1.8 | Lipid metabolism |
| ACD | K09479 | Up | 1.4 | Lipid metabolism |
| ACO | K00232 | Down | -1.5 | Lipid metabolism |
| ACSL | K01897 | Up | 1.2 | Lipid metabolism |
| ApoA-I | K08757 | Down | -2.5 | Lipid metabolism |
| ApoA-IV | K08760 | Down | -1.6 | Lipid metabolism |
| ApoE | K04524 | Down | -1.2 | Lipid metabolism |
| CPT I | K08765 | Up | 1.1 | Lipid metabolism |
| DGAT | K11155 | Up | 1.5 | Lipid metabolism |
| ECH | K01692 | Up | 2.6 | Lipid metabolism |
| FABP | K08750 | Down | -1.2 | Lipid metabolism |
| FATP | K08745 | Down | -1.6 | Lipid metabolism |
| GPD | K00006 | Up | 1.0 | Lipid metabolism |
| HADH | K07515 | Up | 2.6 | Lipid metabolism |
| MDH | K00025 | Up | 1.6 | Lipid metabolism |
| MTTP | K14463 | Down | -1.0 | Lipid metabolism |
| AP-1 | K12393 | Down | -7.6 | Autophagy |
| AP-3 | K12396 | Up | 1.5 | Autophagy |
| ATG4 | K08342 | Down | -1.6 | Autophagy |
| ATG12 | K08336 | Down | -1.2 | Autophagy |
| ATG13 | K08331 | Down | -1.3 | Autophagy |
| ATG14 | K17889 | Up | 2.3 | Autophagy |
| ATPeV | K03661 | Down | -2.1 | Autophagy |
| CTSB | K01363 | Up | 1.4 | Autophagy |
| CTSL | K01365 | Down | -2.0 | Autophagy |
| FGE | K13444 | Down | -1.1 | Autophagy |
| FIP200 | K17589 | Down | -1.1 | Autophagy |
| Jummy | K18086 | Up | 1.0 | Autophagy |
| MTMR3 | K18082 | Up | 2.9 | Autophagy |
| NBR1 | K17987 | Up | 7.1 | Autophagy |
| Rab7 | K07897 | Down | -1.4 | Autophagy |
| ULK1 | K21357 | Down | -1.5 | Autophagy |
| VMP1 | K21248 | Down | -1.3 | Autophagy |
| VPS15 | K08333 | Down | -1.2 | Autophagy |
| VPS34 | K00914 | Down | -6.7 | Autophagy |
