# Supplementary material for: FXR-mediated inhibition of autophagy contributes to FA-induced TG accumulation and accordingly reduces FA-induced lipotoxicity
Source: Cell Commun Signal. 2020 Mar 20;18:47. doi: 10.1186/s12964-020-0525-1 (PMC7082988; doi:10.1186/s12964-020-0525-1)
Supplement: Supplementary file 8 — Additional file 7: Supplemental Fig. S1. Scatter plots showing the correlation between the gene expression profiles of adequate-fat (AF, control) and high-fat (HF, treatment) groups. X-axis and Y -axis mean log2 value of gene expression. Differentially expressed genes are indicated in red (up-regulated expression) and blue (down-regulated expression). Brown means genes that were not differentially expressed. [file 12964_2020_525_MOESM7_ESM.doc]

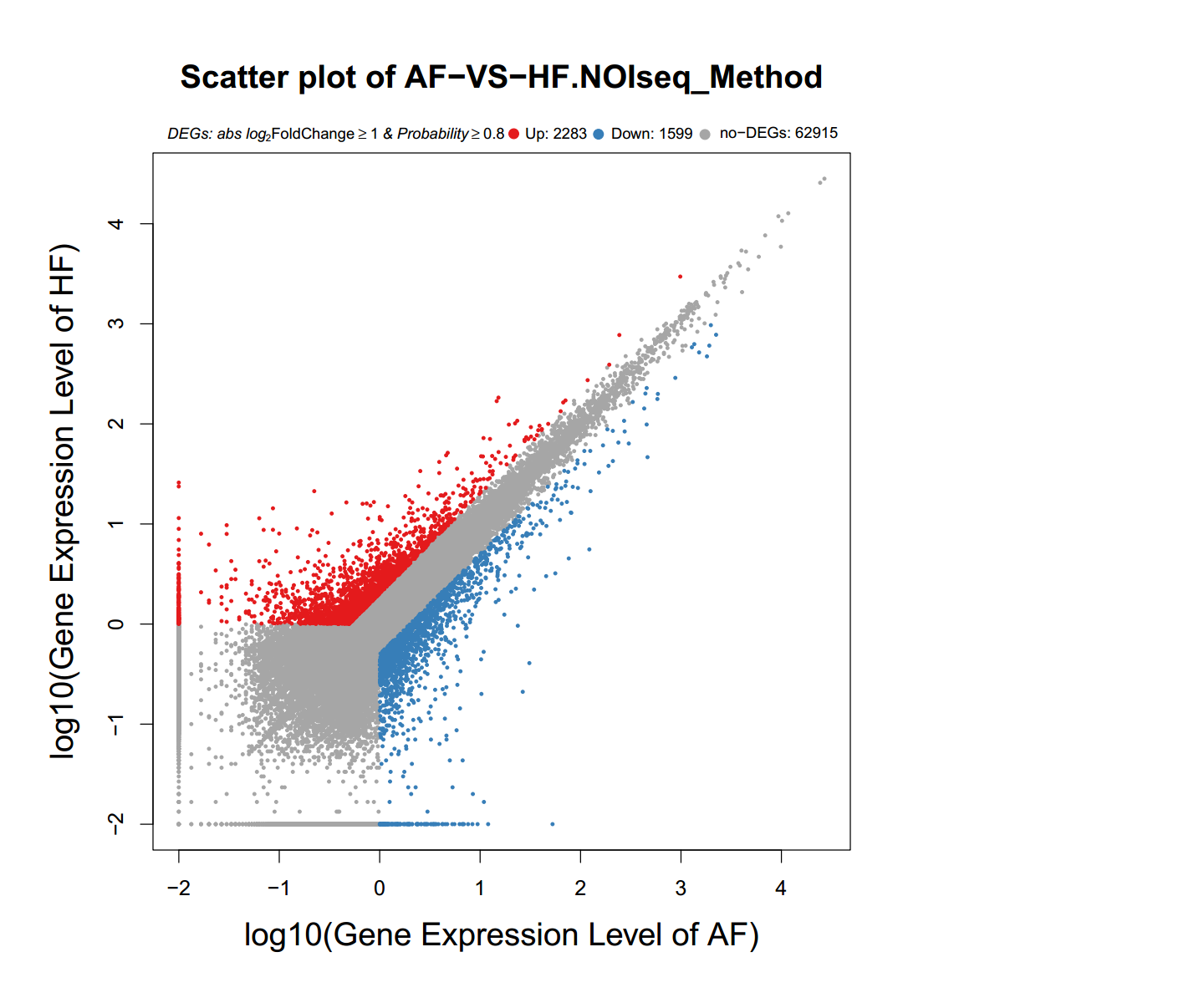


**Supplemental Fig. S1** Scatter plots showing the correlation between the gene expression profiles of adequate-fat (AF, control) and high-fat (HF, treatment) groups. X-axis and Y -axis mean log2 value of gene expression. Differentially expressed genes are indicated in red (up-regulated expression) and blue (down-regulated expression). Brown means genes that were not differentially expressed.
