# Supplementary material for: FXR-mediated inhibition of autophagy contributes to FA-induced TG accumulation and accordingly reduces FA-induced lipotoxicity
Source: Cell Commun Signal. 2020 Mar 20;18:47. doi: 10.1186/s12964-020-0525-1 (PMC7082988; doi:10.1186/s12964-020-0525-1)
Supplement: Supplementary file 9 — Additional file 8: Supplemental Fig. S2. GO functional classification of DEGs. X axis means number of DEGs (the number is presented by its square root value). Y axis represents GO terms. All GO terms are grouped in to three ontologies: red is for biological process, blue is for cellular component and green is for molecular function. [file 12964_2020_525_MOESM8_ESM.doc]

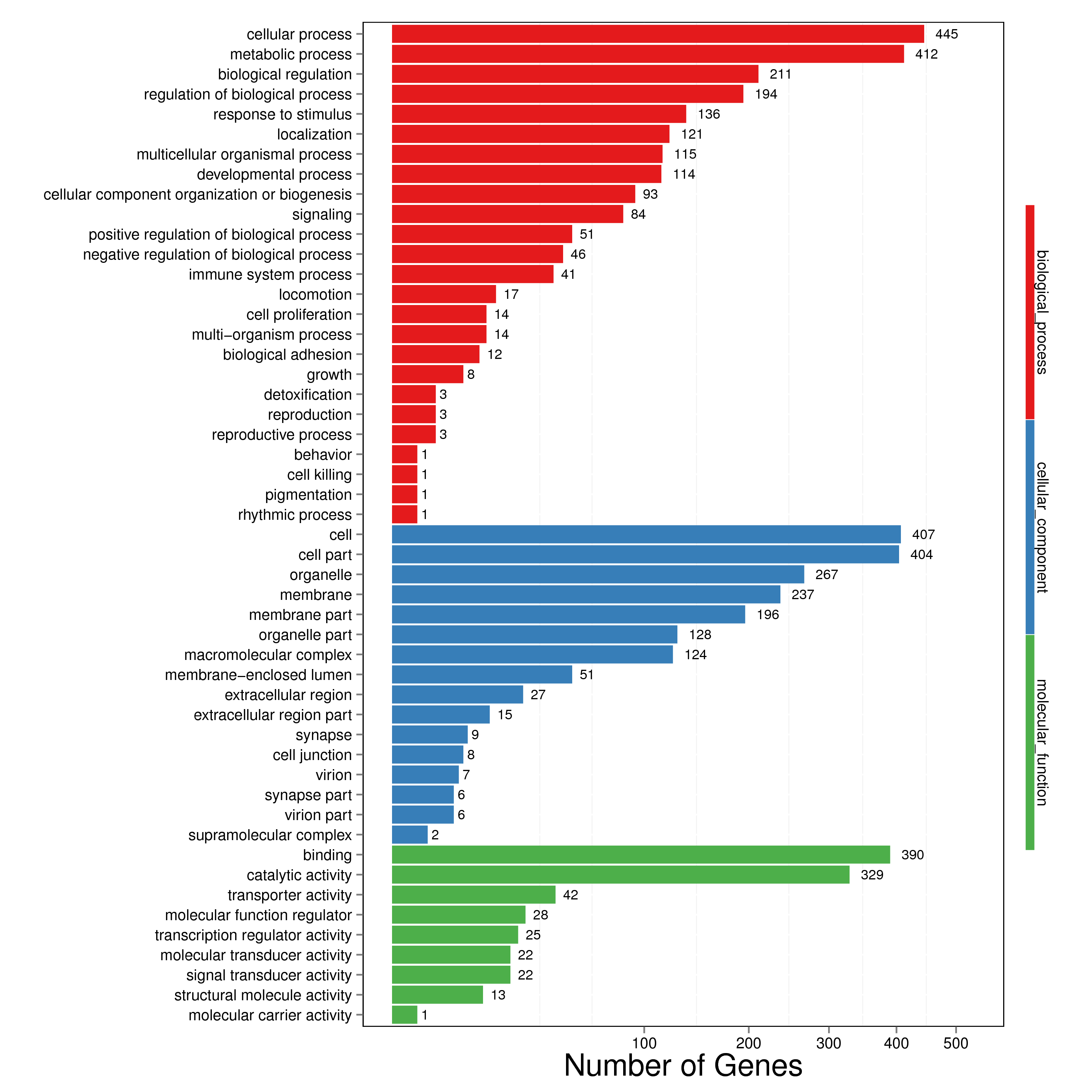


**Supplemental Fig. S2** GO functional classification of DEGs. X axis means number of DEGs (the number is presented by its square root value). Y axis represents GO terms. All GO terms are grouped in to three ontologies: red is for biological process, blue is for cellular component and green is for molecular function.
