# Supplementary material for: FXR-mediated inhibition of autophagy contributes to FA-induced TG accumulation and accordingly reduces FA-induced lipotoxicity
Source: Cell Commun Signal. 2020 Mar 20;18:47. doi: 10.1186/s12964-020-0525-1 (PMC7082988; doi:10.1186/s12964-020-0525-1)
Supplement: Supplementary file 10 — Additional file 9: Supplemental Fig. S3. KEGG functional classification of DEGs. X axis means number of DEGs. Y axis represents the second KEGG pathway terms, and then the second pathway terms are grouped in the top pathway terms as indicated in different color. [file 12964_2020_525_MOESM9_ESM.doc]

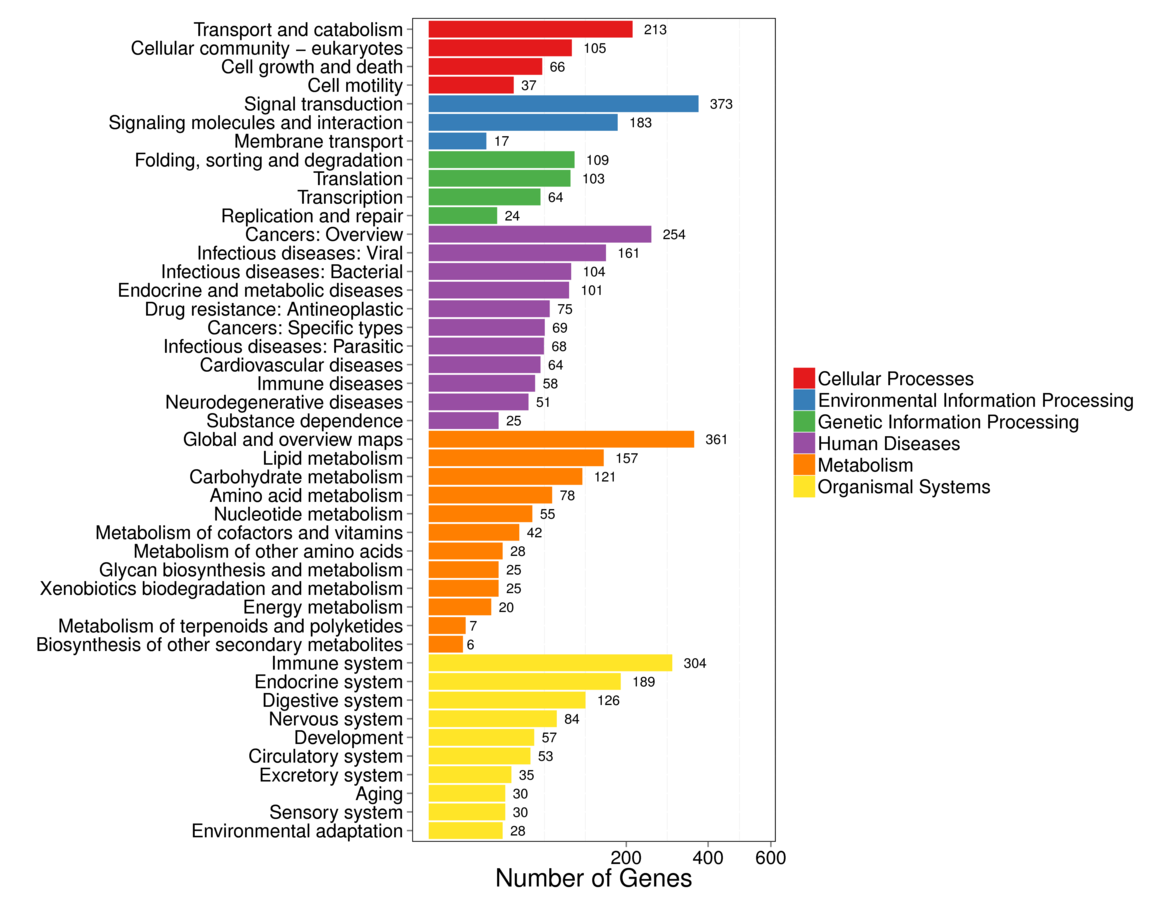


**Supplemental Fig. S3** KEGG functional classification of DEGs. X axis means number of DEGs. Y axis represents the second KEGG pathway terms, and then the second pathway terms are grouped in the top pathway terms as indicated in different color.
