# Supplementary material for: FXR-mediated inhibition of autophagy contributes to FA-induced TG accumulation and accordingly reduces FA-induced lipotoxicity
Source: Cell Commun Signal. 2020 Mar 20;18:47. doi: 10.1186/s12964-020-0525-1 (PMC7082988; doi:10.1186/s12964-020-0525-1)
Supplement: Supplementary file 11 — Additional file 10: Supplemental Fig. S4. Comparison of mRNA levels between RNA-seq and Q-PCR results. The y-axis is the gene expressed fold change and the x-axis is the gene name. The correlation coefficient between RNA-seq and Q-PCR results was 0.903 (p < 0.001). [file 12964_2020_525_MOESM10_ESM.doc]

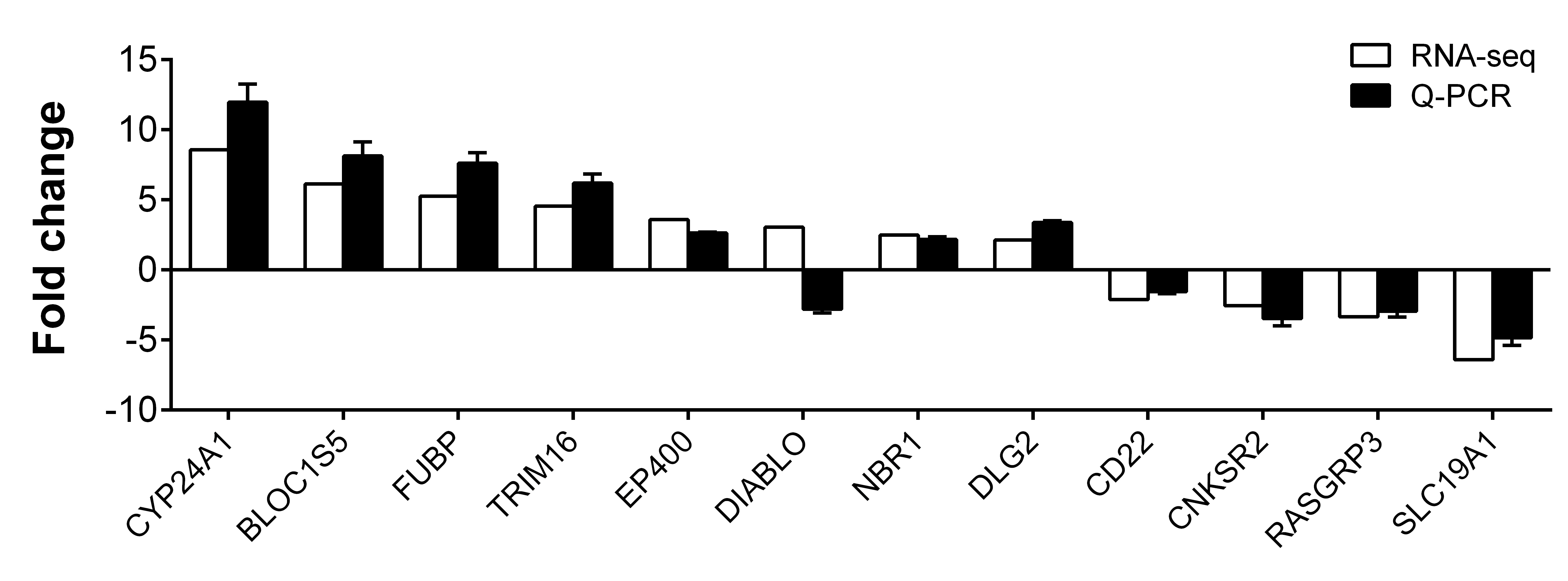


**Supplemental Fig. S4** Comparison of mRNA levels between RNA-seq and Q-PCR results. The y-axis is the gene expressed fold change and the x-axis is the gene name. The correlation coefficient between RNA-seq and Q-PCR results was 0.903 (p < 0.001).
