# Supplementary material for: FXR-mediated inhibition of autophagy contributes to FA-induced TG accumulation and accordingly reduces FA-induced lipotoxicity
Source: Cell Commun Signal. 2020 Mar 20;18:47. doi: 10.1186/s12964-020-0525-1 (PMC7082988; doi:10.1186/s12964-020-0525-1)
Supplement: Supplementary file 12 — Additional file 11: Supplemental Fig. S5. Effects of FA and rapamycin (autophagy agonist) incubation on autophagy in yellow catfish hepatocytes at 48 h. A) Representative confocal microscopic image of hepatocytes co-stained with MDC and LysoTracker. B) Representative confocal microscopic image of hepatocytes stained with AO. C) Flow cytometric analysis of Bodipy, LysoTracker and AO staining. RM, rapamycin. FA, oleic and palmitic acid at a ratio of 1:1. Values are means ± SEM (n = 3). Asterisks (∗) indicate significant differences between the two groups (p < 0.05). [file 12964_2020_525_MOESM11_ESM.doc]

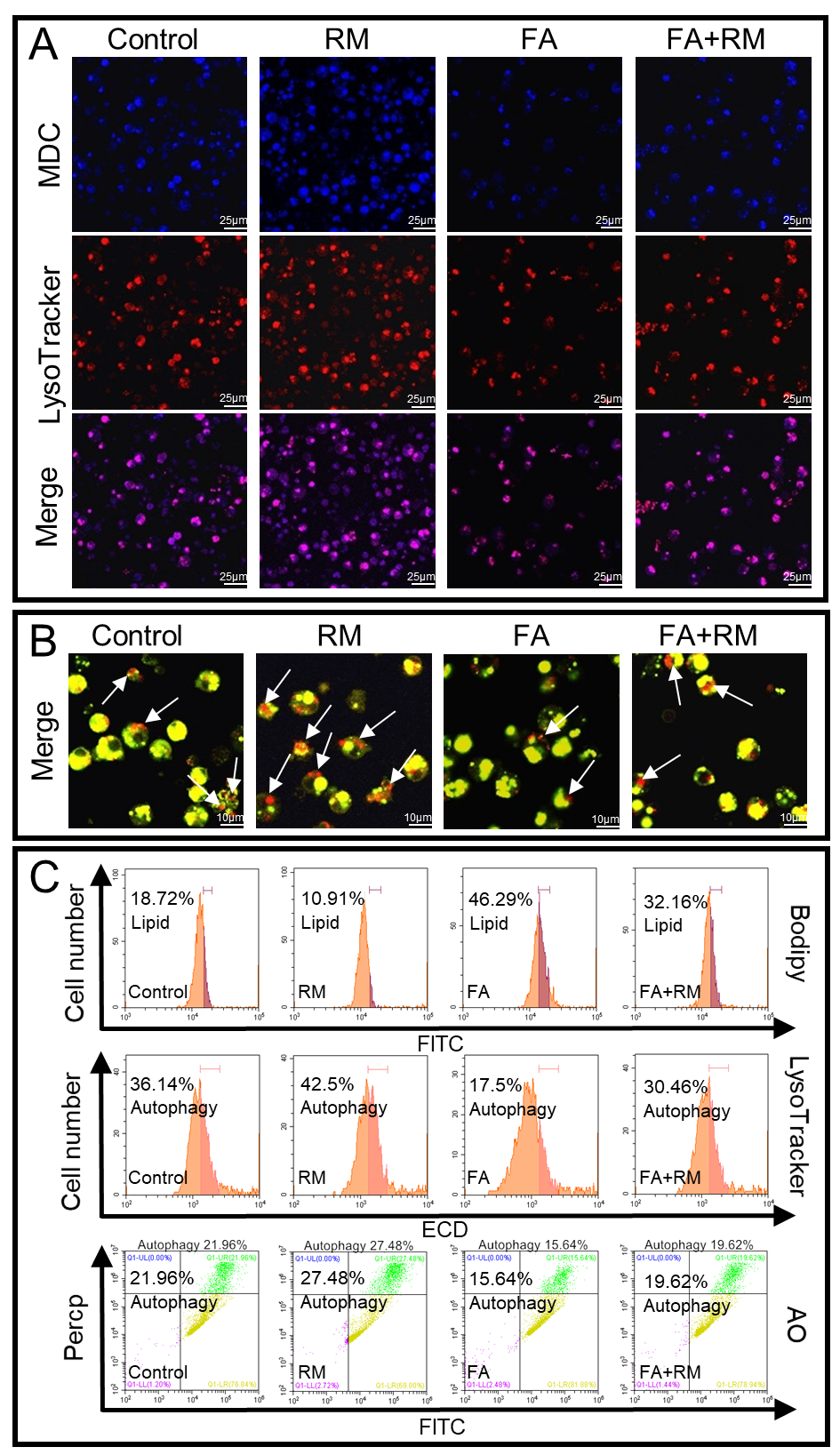


**Supplemental Fig. S5** Effects of FA and rapamycin (autophagy agonist) incubation on autophagy in yellow catfish hepatocytes at 48 h. A) Representative confocal microscopic image of hepatocytes co-stained with MDC and LysoTracker. B) Representative confocal microscopic image of hepatocytes stained with AO. C) Flow cytometric analysis of Bodipy, LysoTracker and AO staining. RM, rapamycin. FA, oleic and palmitic acid at a ratio of 1:1. Values are means ± SEM (n=3). Asterisks (∗) indicate significant differences between the two groups (*p* < 0.05).
