# Supplementary material for: FXR-mediated inhibition of autophagy contributes to FA-induced TG accumulation and accordingly reduces FA-induced lipotoxicity
Source: Cell Commun Signal. 2020 Mar 20;18:47. doi: 10.1186/s12964-020-0525-1 (PMC7082988; doi:10.1186/s12964-020-0525-1)
Supplement: Supplementary file 14 — Additional file 13: Supplemental Fig. S7. Effects of FA and 3-methyladenine (autophagy inhibitor) on autophagy in yellow catfish hepatocytes at 48 h. A) Flow cytometric analysis and relative mean fluorescence intensity of LysoTracker and AO staining. B) Expression of genes involved in autophagy. MA, 3-methyladenine. FA, oleic and palmitic acid at a ratio of 1: 1. Values are means ± SEM (n = 3). Asterisks (∗) indicate significant differences between the two groups (p < 0.05). [file 12964_2020_525_MOESM13_ESM.doc]

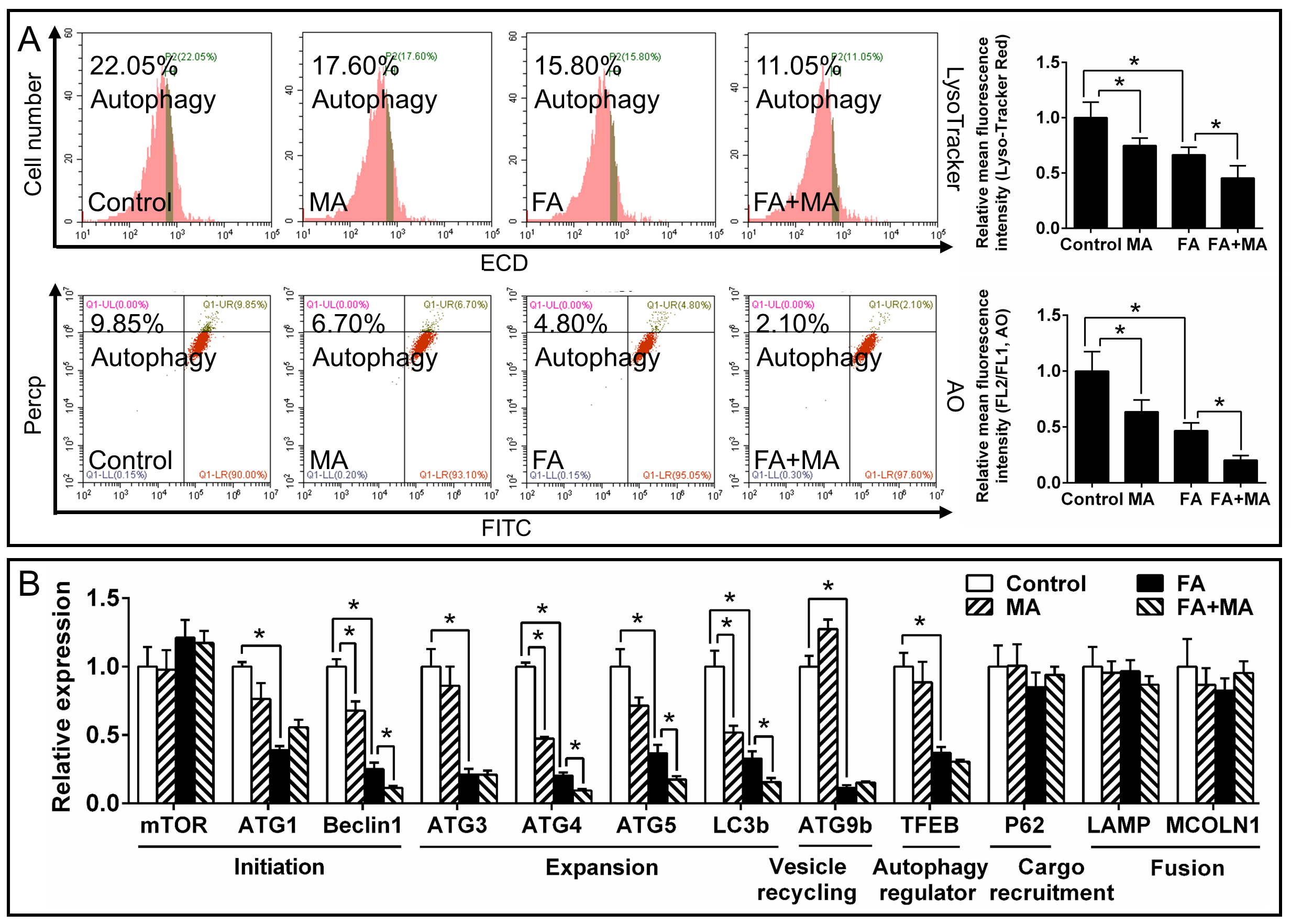


**Supplemental Fig. S7** Effects of FA and 3-methyladenine (autophagy inhibitor) on autophagy in yellow catfish hepatocytes at 48 h. A) Flow cytometric analysis and relative mean fluorescence intensity of LysoTracker and AO staining. B) Expression of genes involved in autophagy. MA, 3-methyladenine. FA, oleic and palmitic acid at a ratio of 1: 1. Values are means ± SEM (n=3). Asterisks (∗) indicate significant differences between the two groups (*p* < 0.05).
