# Supplementary material for: FXR-mediated inhibition of autophagy contributes to FA-induced TG accumulation and accordingly reduces FA-induced lipotoxicity
Source: Cell Commun Signal. 2020 Mar 20;18:47. doi: 10.1186/s12964-020-0525-1 (PMC7082988; doi:10.1186/s12964-020-0525-1)
Supplement: Supplementary file 15 — Additional file 14: Supplemental Fig. S8. Effects of FA and 3-methyladenine (autophagy inhibitor) incubation on lipid deposition and metabolism in yellow catfish hepatocytes at 48 h. A) Flow cytometric analysis of Bodipy staining. B) Contents of TG and NEFA, and enzymatic activities. C) Expression of genes involved in lipid metabolism. MA, 3-Methyladenine. FA, oleic and palmitic acid at a ratio of 1:1. Values are means ± SEM (n = 3). Asterisks (∗) indicate significant differences between the two groups (p < 0.05). [file 12964_2020_525_MOESM14_ESM.doc]

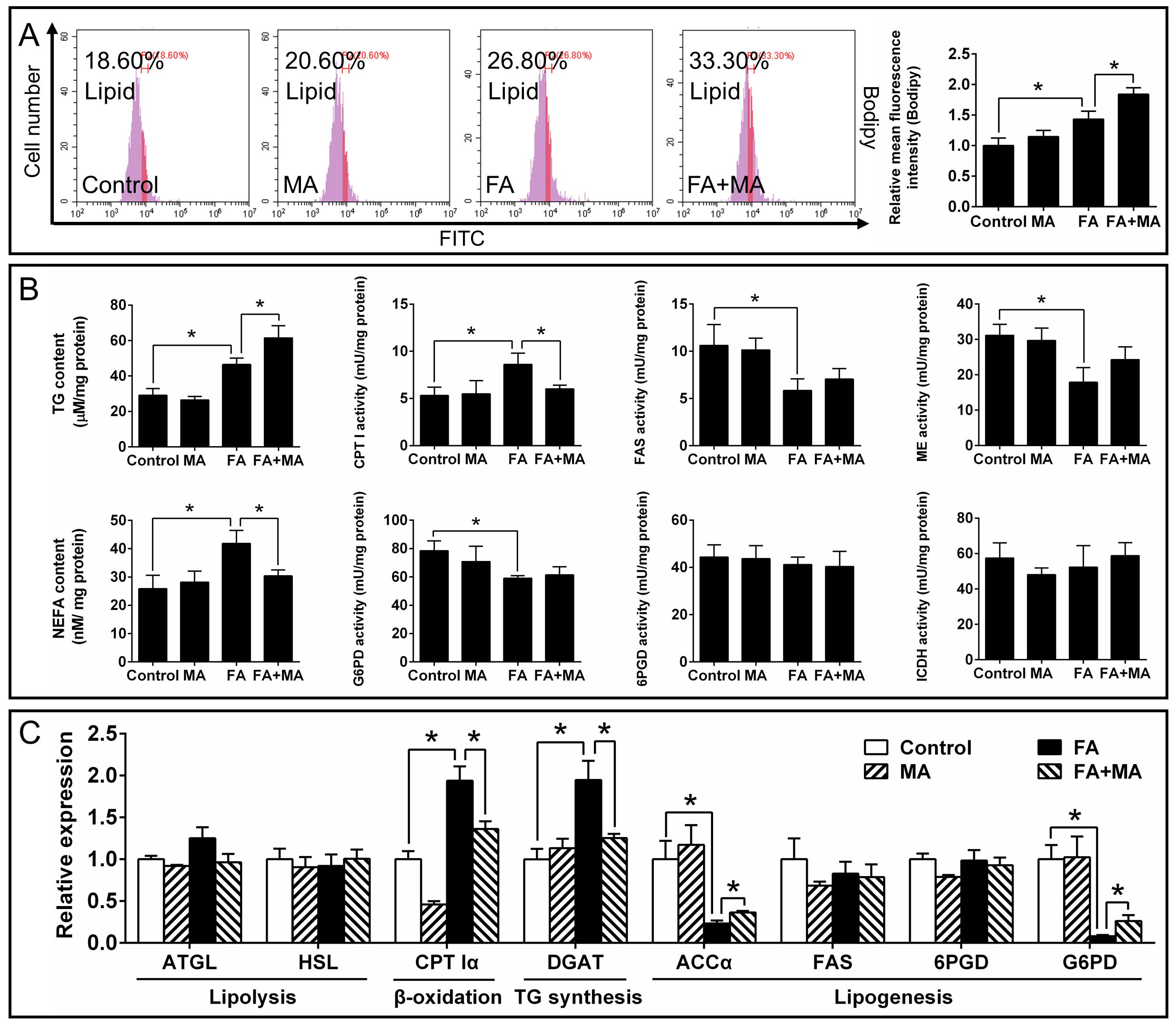


**Supplemental Fig. S8** Effects of FA and 3-methyladenine (autophagy inhibitor) incubation on lipid deposition and metabolism in yellow catfish hepatocytes at 48 h. A) Flow cytometric analysis of Bodipy staining. B) Contents of TG and NEFA, and enzymatic activities. C) Expression of genes involved in lipid metabolism. MA, 3-Methyladenine. FA, oleic and palmitic acid at a ratio of 1:1. Values are means ± SEM (n=3). Asterisks (∗) indicate significant differences between the two groups (*p* < 0.05).
