# Supplementary material for: FXR-mediated inhibition of autophagy contributes to FA-induced TG accumulation and accordingly reduces FA-induced lipotoxicity
Source: Cell Commun Signal. 2020 Mar 20;18:47. doi: 10.1186/s12964-020-0525-1 (PMC7082988; doi:10.1186/s12964-020-0525-1)
Supplement: Supplementary file 16 — Additional file 15: Supplemental Fig. S9. Effect of FA and guggulsterone (FXR inhibitor) incubation on apoptosis in yellow catfish hepatocytes at 48 h. A) Flow cytometric analysis of apoptosis. B) caspase 3 activity, cell viability and quantitation of relative mean fluorescence intensity of apoptosis. GS, guggulsterone. FA, oleic and palmitic acid at a ratio of 1:1. Values are means ± SEM (n = 3). Asterisks (∗) indicate significant differences between the two groups (p < 0.05). [file 12964_2020_525_MOESM15_ESM.doc]

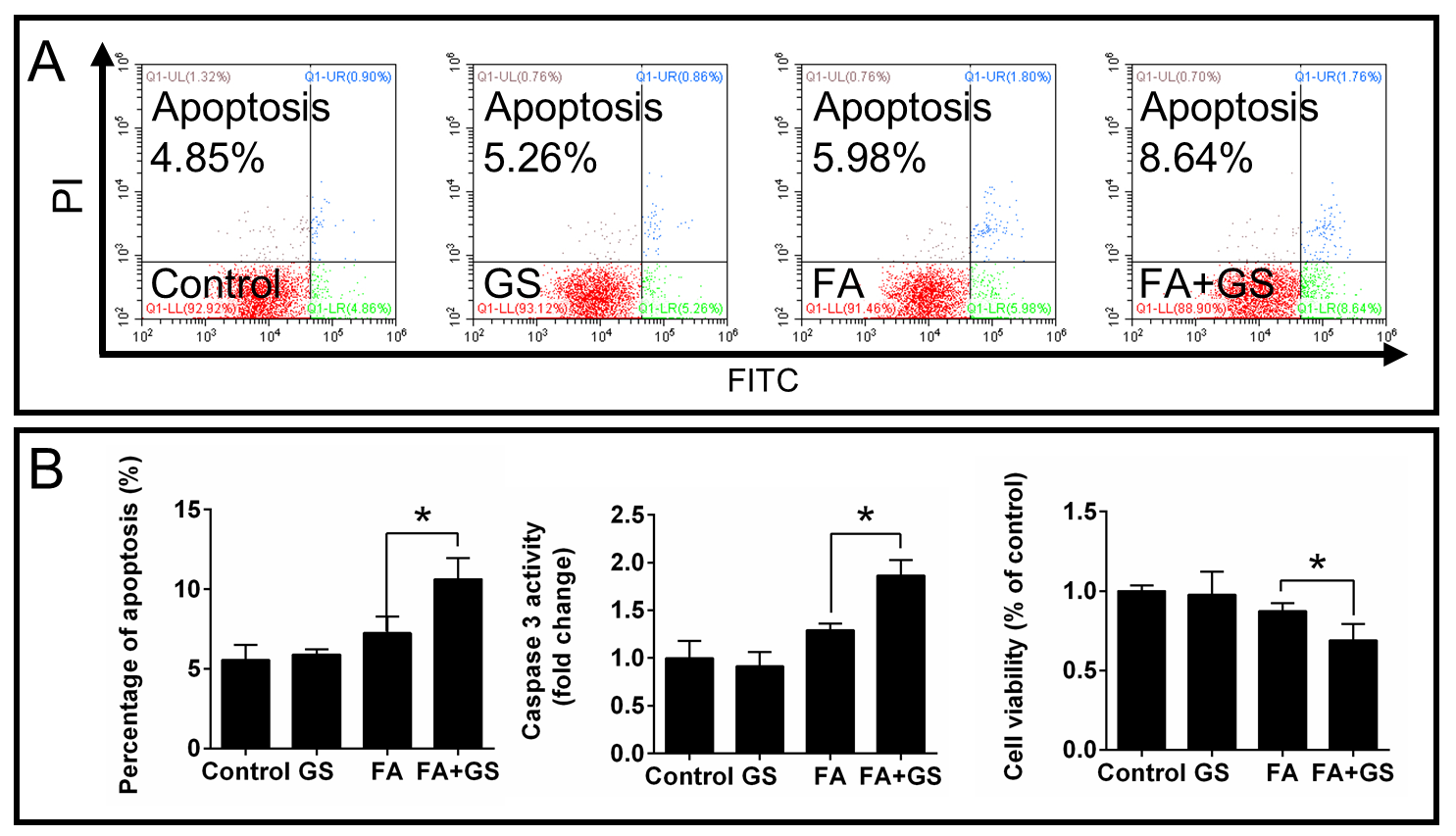


**Supplemental Fig. S9** Effect of FA and guggulsterone (FXR inhibitor) incubation on apoptosis in yellow catfish hepatocytes at 48 h. A) Flow cytometric analysis of apoptosis. B) caspase 3 activity, cell viability and quantitation of relative mean fluorescence intensity of apoptosis. GS, guggulsterone. FA, oleic and palmitic acid at a ratio of 1:1. Values are means ± SEM (n=3). Asterisks (∗) indicate significant differences between the two groups (*p* < 0.05).
